# Conservation and sustainable use of the medicinal Leguminosae plants from Angola

**DOI:** 10.7717/peerj.6736

**Published:** 2019-05-23

**Authors:** Silvia Catarino, Maria Cristina Duarte, Esperança Costa, Paula Garcia Carrero, Maria M. Romeiras

**Affiliations:** 1 Linking Landscape, Environment, Agriculture and Food (LEAF), Instituto Superior de Agronomia (ISA), Universidade de Lisboa, Lisboa, Portugal; 2 Forest Research Center (CEF), Instituto Superior de Agronomia (ISA), Universidade de Lisboa, Lisboa, Portugal; 3 Centre for Ecology, Evolution and Environmental Changes (cE3c), Faculdade de Ciências, Universidade de Lisboa, Lisboa, Portugal; 4 Centro de Botânica, Universidade Agostinho Neto, Luanda, Angola

**Keywords:** Ethnobotany, Conservation, Fabaceae, Timber species, Southern Africa

## Abstract

Leguminosae is an economically important family that contains a large number of medicinal plants, many of which are widely used in African traditional medicine. Angola holds a great socio-cultural diversity and is one of the richest floristic regions of the world, with over 900 native Leguminosae species. This study is the first to assess the medicinal uses of the legumes in Angola and provides new data to promote the conservation and the sustainable use of these unique resources. We document the ethnobotanical knowledge on Angola by reviewing the most important herbarium collections and literature, complemented by recent field surveys. Our results revealed that 127 native legume species have medicinal uses and 65% of them have other important uses by local populations. The species with most medicinal applications are *Erythrina abyssinica, Bauhinia thonningii* and *Pterocarpus angolensis*. The rich flora found in Angola suggests an enormous potential for discovery of new drugs with therapeutic value. However, the overexploitation and the indiscriminate collection of legumes for multiple uses such as forage, food, timber and medical uses, increases the threats upon the native vegetation. Efforts to assess the conservation status of these species are urgently needed, and future actions should promote the sustainable use of medicinal plants in Angola together with the implementation of conservation strategies.

## Introduction

Over the past decades, following the United Nations Conference on Environment and Development (UNCED) held in Rio de Janeiro in 1992, the conservation of biodiversity has come to be understood as an essential aspect of sustainable development worldwide (Najam & Cleveland, 2005). In particular, Africa’s growing population, which is set to nearly double by 2050, and the implications for food production and provision for a growing population while maintaining healthy ecosystems and habitats, have become one of the most pressing issues of the 21st century (Thorn et al., 2015).

Two-thirds of all angiosperm species are found within the tropics (Pimm & Joppa, 2015). It is now well-established that plants supports critical ecosystem services, which includes: (i) supporting services (e.g., nutrient cycling, and primary production); (ii) regulating services (e.g., climate regulation, and pollination services); (iii) provisioning services (e.g., fuel wood, edible, medicinal, and aromatic plants); and (iv) cultural services (e.g., education, recreational, tourism, bequest or aesthetic value) (Millennium Ecosystem Assessment, 2005). In sub-Saharan Africa, the majority of people depend mainly on natural resources for subsistence and income generation and urgent actions are required to achieve the global goals to reduce the rate of biodiversity loss, degradation of ecosystems and subsequent reduction in goods and services that can be obtained from it (Mertz et al., 2007). In this region, medicinal plants are one of the most important elements of biodiversity, because of their role in ecosystem services such as healthcare, cultural value and heritage, local economics and human well-being (Okigbo, Eme & Ogbogu, 2008). Indigenous knowledge about the use of plants in traditional medicine constitutes a strategic resource and several studies have highlighted the great potential of native medicinal plants for therapeutic purposes (for reviews see: Van Wyk, 2008; Moyo, Aremu & Van Staden, 2015; Máthé, Neffati & Najjaa, 2017). Moreover, much of our understanding is still anchored in indigenous knowledge rather than in scientific studies. Hence, preserving this traditional knowledge is a critical aspect of conservation efforts focusing on useful plant species, especially given the risk that this knowledge could be lost in future generations. Furthermore, the medicinal plants are being overexploited in sub-Saharan Africa, and the high rates of destruction of their natural habitat is harmful not only to single species, but also to whole communities and ecosystems (Okigbo, Eme & Ogbogu, 2008). The consequences of these threats are such that they demand our urgent attention to conserve wild plant species used in African traditional medicine. In order to achieve this objective, reliable data are needed on their distribution and level of use, especially in understudied regions.

Angola is the largest country in southern Africa (1,246.700 km^2^) with an estimated population of over 26 million, according to the 2014 population census (INE, 2014). The country is divided into eighteen provinces (Data S1) and presents a great ethnical diversity with about 90 different groups, each with its own culture, beliefs and ways of appropriating nature (Schubert, 2017). The largest ethnic groups are the Ovimbundu and Ambundu, with ca. 37% and 25% of the total population respectively. Other representative groups are Bakongo, Chokwe, Ovambo, Ganguela and Xindonga (Gosoniu, Veta & Vounatsou, 2010).

Angola encompasses a very wide range of ecosystems and habitats, and according to the World Wildlife Fund (WWF) (Olson et al., 2001; Burgess et al., 2004) there are seven major terrestrial biomes: (1) tropical and subtropical moist broadleaf forests; (2) tropical and subtropical dry broadleaf forests; (3) tropical and subtropical grasslands, savannas, and shrublands; (4) flooded grasslands and savannas; (5) montane grasslands and shrublands; (6) deserts and xeric shrublands; and (7) mangroves (more details are provided in Data S2). Although there is a wide-range of biomes, substantial habitat loss is confirmed by recent studies, above all with regard to the dry forests that are currently experiencing the consequences of human exploitation on an unprecedented scale (see for more details: Hansen et al., 2013; Romeiras et al., 2014). Without a clear involvement of local organizations to halt the risks associated with the threat to the country’s biodiversity, progressive ecological disturbance may lead to the extinction of species with an inherent value and potential for agriculture, forestry or medicinal purposes.

Traditional medicine plays an important role in health care in Angola (Costa & Pedro, 2013), and since the middle of the 20th century some documents were published containing ethnobotanical information (e.g.,  Gossweiler, 1953; Peres, 1959; Santos, 1967; Santos, 1989). At the end of the 20th century, Bossard (1996) published the most comprehensive study, and a total of 780 species used in traditional medicine were recorded. Over the last decade, the study of Angola’s medicinal flora has been the subject of more research, namely by Costa & Pedro (2013), Urso, Signorini & Bruschi (2013), Göhre et al. (2016), Bruschi et al. (2017) and Heinze et al. (2017).

Angola is one of the richest floristic regions of Africa, with over 6,700 native plant species, with ca. 15% Leguminosae species which thus form the richest family in this country (Figueiredo & Smith, 2008). This family encompasses key-crops, medicinal plants and important African timber species (Soares et al., 2007) thus making it an important family of flowering plants with economic and medicinal applications. The uses of legumes as a source of drugs have been extensively reported in several studies (e.g.,  Graham & Vance, 2003; Howieson et al., 2008). For instance, *Acacia senegal* (L.) Willd., also known as gum Arabic, is native in arid regions of sub-Saharan Africa, and is widely used as a food additive (e.g., in commercial emulsification for the production of beverages and flavor concentrates) and in the pharmaceutical industry, namely to treat bacterial and fungal infections of the skin and mouth (Mahomoodally, 2013). Current evidence suggests that medicines derived from several Leguminosae species have important therapeutic effects in cancer treatments. The methanol extract of the bark of *Guibourtia tessmannii* (Harms) J.Leonard (from Cameroon) has shown antiproliferative activity against cervical cancer cells (Kuete et al., 2013), while serine protease inhibitors from *Cajanus cajan* (L.) Millsp. seeds (also known as the pigeon pea) demonstrated anticancer potential (Shamsi et al., 2017).

As stated above, Angola hosts high levels in terms of the species richness and endemism, but threats to this rich flora and their habitats are emerging. Therefore, it is imperative to conserve and study its biodiversity, also with regard to useful plant species. In particular, legumes are a highly appropriate *proxy* group for an understanding of the diversity and conservation issues of useful plants as a whole, in view of the fact that it (a) forms the largest plant family in Angola (Figueiredo & Smith, 2008), (b) has diversified in almost all biomes and ecoregions and is often a dominant component of the major habitats (Olson et al., 2001), (c) is also of ecological importance in maintaining soil fertility through fixation of atmospheric nitrogen by bacteria in nodules on their roots (LPWG, 2017) and (d) is known to contain a wide range of uses including many commercially important species (Soares et al., 2007).

Therefore, this study focuses on the knowledge and use of the flora as a major Angolan socio-cultural heritage, and particularly the diverse Leguminosae family, aiming to identify the species used in traditional medicine. A better understanding of the multiple uses of these medicinal plants, including food and timber, will provide key knowledge to conserve plant diversity in Angola and tackle the potential threats that are endangering these species’ survival. In particular, this study seeks to respond to two central questions: (i) which Leguminosae species are used in traditional medicine, and (ii) what can be done to ensure the conservation and sustainable use of these medicinal species in Angola?

## Materials & Methods

### Data collection

Data on the Leguminosae plant species used in the traditional medicine in Angola was obtained by means of a comprehensive review conducted through the study of numerous herbarium specimens, and of published works and online databases. To provide a critical and updated review of Angola’s medicinal plants, interviews with indigenous healers were conducted over the last two decades in some regions of Angola.

Therefore, this study was made using four main sources:

1. The Angolan collections held in the Herbarium of the Instituto de Investigação Científica Tropical, University of Lisbon (LISC). This is the most comprehensive and representative collection of the Angolan flora comprising over 80,000 specimens that have been collected since the 19th century. Information recorded on the labels allowed us to get data on the medicinal and other uses (e.g., food, timber, fibers and forage), plant parts used, diseases treated, as well as growth form and distribution of each species within Angola.
2. A thorough investigation of the medicinal plant data described in literature. We review data available from the past (e.g.,  Ficalho, 1947; Gossweiler, 1953; Peres, 1959; Santos, 1967; Santos, 1989; Van-Dúnem, 1994; Bossard, 1996) and also more contemporary sources (e.g.,  Costa, Dombo & Paula, 2009; Leyens & Lobin, 2009; Costa & Pedro, 2013; Göhre et al., 2016; Bruschi et al., 2017; Heinze et al., 2017). Furthermore, species occurrences were compiled from specialized bibliography, namely in *Conspectus Florae Angolensis* (e.g.,  Exell & Mendonça, 1956; Exell & Fernandes, 1962; Exell & Fernandes, 1966).
3. Online databases, namely: (i) PROTA - The Plant Resources of Tropical Africa (https://www.prota4u.org/), which has detailed information on the taxonomy and uses of many African plants; (ii) IUCN - Red List of Threatened Species (http://www.iucnredlist.org) that provides useful information on each species assessed, threats and conservation actions; and (iii) Global Biodiversity Information Facility (GBIF) to get distribution data.
4. Field research carried out by one of the authors (E. Costa), which substantially contributed to updating and complementing the inventory of Angola’s medical flora. Field surveys were conducted in four provinces of Angola: Luanda, Bengo, Zaire and in the exclave province of Cabinda, located in the north of Angola (more information is provided in Data S1). In each rural community, a first meeting was held with the village chief (Soba –Portuguese name) and his advisors to inform them about the purposes of our work and get their support for the choice of the best skilled traditional healers in each community. The ethnobotanical surveys were conducted using semi-structured questionnaires, previously prepared and tested (Albuquerque et al., 2014). During the interviews each healer was previously informed about the objectives of the study and asked to get his informed consent to participate.

In the course of fieldwork, botanical samples were collected for herbarium vouchers. These specimens were preserved for later identification and are kept in the LISC Herbarium and LUA Herbarium (University of Agostinho Neto, Luanda Angola).

### Database construction and calculation of pharmacological importance

Based on the best currently available knowledge, we combined ethnobotanical data to complete a database with all the Leguminosae medicinal species known from Angola. This database includes for each species the scientific name and subfamily; distribution in Angola; ethnobotanical data (e.g., plant part(s) used; group of diseases; and other uses such as food, timber, fibers and forage); and their conservation status and main threats, using the Threats Classification Scheme version 3.2 proposed by IUCN (Data S3) (http://www.iucnredlist.org/technical-documents/classification-schemes/threats-classification-scheme). Moreover, for each species it is indicated the number of different sources (e.g., herbarium specimens, published sources, and fieldwork) that refers to its medicinal uses, as a measure to provide more confidence in the legitimacy of usage data, and estimate the ethnobotanical importance of the species.

**Table 1:** Medicinal Leguminosae plants of Angola.

**Taxon**	**Subfamilies**	**Native status**	**Main distribution in Angola (Provinces)**^a^	**Important uses**	**Groups of diseases**^b^	**Parts used**^c^	**PI**^d^	**Voucher**^e^	**References**^f^	**Healers information**^g^	**Potential threats**^h^	**Number of information sources**^i^
*Abrus canescens* Baker	Papilionoideae	Native	CN, LN, MA, UI	Medicinal	Z	Rt	0.06	Martins 79	13, 14		3	1
*Abrus precatorius* L.	Papilionoideae	Native	BE, BI, BO, CN, CS, CU, HI, LA, NA	Medicinal, Ornamental	W, Others	Lv, Rt	0.11	Teixeira 476	13	*	5.2	1
*Acacia antunesii* Harms	Caesalpinioideae	Endemic	CU, HI	Medicinal	Others	Lv	0.06	Antunes vel Dekindt 28	3		1	1
*Acacia arenaria* Schinz	Caesalpinioideae	Native	CC, CU, HI, NA	Medicinal, Forage, Fiber	T	Rt	0.06	Menezes 925	3, 13		2.3, 5.2	1
*Acacia brevispica* Harms	Caesalpinioideae	Native	BI, CU, HI, NA	Medicinal, Timber, Forage, Honey plant, Fiber	C, D, I, Q	Lv	0.22	Correia 1165	6, 13, 14		2.3, 5.3	3
*Acacia goetzei* Harms	Caesalpinioideae	Native	BE, HI, MA, NA	Medicinal, Timber	C	Lv, Rt	0.06	Henriques 607	11, 13		5.3	1
Acacia karroo Hayne	Caesalpinioideae	Native	HI	Medicinal, Forage, Fiber	A, N	Rt	0.11	Dechamps, Murta & Silva 1235	6			1
*Acacia kirkii* Oliv. subsp. *kirkii*	Caesalpinioideae	Native	CU, HI, NA	Medicinal, Forage, Gum	A, N	Rt	0.11	Torre 8704	6, 13			1
*Acacia kirkii* subsp. *mildbraedii* (Harms) Brenan	Caesalpinioideae	Native	HI	Medicinal	N, Others		0.11	Barbosa 10674	13, 14			1
*Acacia reficiens* Wawra	Caesalpinioideae	Native	BE, NA	Medicinal, Timber, Forage, Fiber	C, J, Others	Rt, St	0.17	Gossweiler 12847	6, 13		2.3, 5.3	1
*Acacia sieberiana* DC.	Caesalpinioideae	Native	BE, BO, CC, CN, CS, CU, HA, HI, MA, NA	Medicinal, Food, Timber, Forage, Gum	E, H, J, Q, W, Others	Lv, Rt, St	0.33	Murta & Silva 744	6, 7, 13	*	2.3, 5.3	3
*Acacia welwitschii* Oliv.	Caesalpinioideae	Endemic	BE, BO, CN, CS, CU, HI, LA	Medicinal, Food	B, T	Bk, Rt	0.11	Barbosa 10974	14	*	5.3	2
*Adenocarpus mannii* (Hook.f.) Hook.f.	Papilionoideae	Native	BE, HI, NA	Medicinal, Timber, Forage, Tanning	P		0.06	Gossweiler 12400	6, 13		5.3	1
*Adenodolichos anchietae* (Hiern) Harms	Papilionoideae	Native	BI, CC, CS, HI	Medicinal	B, D, N, T		0.22	Mendes 2445	6			1
*Adenodolichos rhomboideus* (O.Hoffm.) Harms	Papilionoideae	Native	BE, CC, CS, HA, HI, MA	Medicinal, Food	B, D, N, T	Fr, St	0.22	Daniel 10	6, 14			1
*Aeschynomene angolense* Rossberg	Caesalpinioideae	Endemic	BE, HA	Medicinal	V		0.06	Moreno 273	6			1
*Aeschynomene fluitans* Peter	Caesalpinioideae	Native	BE, CC, CU, HA, HI	Medicinal	Others		0.06	Henriques 221	6		7, 11	1
*Aeschynomene fulgida* Baker	Caesalpinioideae	Native	BI, BO, CC, HA, HI, LS, MO	Medicinal	B	Rt	0.06	Teixeira 1041	8, 13			1
*Afzelia quanzensis* Welw.	Detarioideae	Native	BE, BI, CA, CC, CN, CS, CU, HI, MA, NA, UI	Medicinal, Food, Timber, Forage, Ornamental, Honey plant	A, G, J, L, Q, T, U, Others	Bk, Rt	0.44	Melo & Conceição 15	8, 13		5.3	1
*Albizia adianthifolia* (Schum.) W.Wight	Caesalpinioideae	Native	BI, BO, CA, CN, CS, HI, LN, LS, MA, MO, UI, ZA	Medicinal, Food, Timber, Forage, Shade	D, T	Lv, Rt	0.11	Teixeira & Andrade 8322	11, 12, 13, 14		2.3, 5.3	1
*Albizia anthelmintica* Brongn.	Caesalpinioideae	Native	CC, CU, HI, NA	Medicinal, Food, Timber, Forage, Ornamental, Erosion control	B, E, T	Bk, Rt	0.17	Menezes 788	6, 13, 14		2.3, 5.3	2
*Albizia antunesiana* Harms	Caesalpinioideae	Native	BE, BI, CC, CN, CS, HA, HI, MA, MO, NA	Medicinal, Timber, Forage, Tanning, Honey plant	D, J, P, R	Bk, Rt	0.22	Gossweiler 12645	6, 7, 8, 13		2.3, 5.3	2
*Albizia gummifera* (J.F.Gmel.) C.A.Sm.	Caesalpinioideae	Native	BE, CN, CS, LN, LS	Medicinal, Timber, Forage, Honey plant	C, E, F, F*, Q, U	Bk, Fr, Lv	0.28	Silva 726	7, 13	*	3, 5.3	2
*Albizia versicolor* Oliv.	Caesalpinioideae	Native	BO, CC, CN, CS, CU, HI, LA, MA, ZA	Medicinal, Timber, Forage, Ornamental, Fiber, Tanning, Honey plant	B	Rt	0.06	Teixeira 2142	6, 13		5.3	1
*Alysicarpus ovalifolius* (Schum.) Leonard	Papilionoideae	Native	CN, MA, ZA	Medicinal, Forage	D	Lv	0.06	Rocha 116	13, 14		2.3	1
*Argyrolobium aequinoctiale* Baker	Papilionoideae	Native	BI, CS, HA, HI, MA, NA	Medicinal	X		0.06	Teixeira 2132	6			1
*Baphia massaiensis* Taub. subsp. *obovata* (Schinz) Brummitt	Papilionoideae	Native	BE, CC, CU, HI, LS, MO, NA	Medicinal, Forage, Toothbrush	J	Fl, Fr, Lv	0.06	Monteiro, Santos & Murta 515	13, 14		2.3	1
*Bauhinia petersiana* Bolle	Cercidoideae	Native	CC, CU, HI, MO	Medicinal, Food, Forage, Honey plant	E	Lv	0.06	Teixeira 1543	6, 7, 13, 14	*	5.2	3
*Bauhinia thonningii* Schum.	Cercidoideae	Native	BE, BI, CC, CN, CS, CU, HA, HI, LN, MA, UI, ZA	Medicinal, Food, Timber, Forage, Fiber, Toothbrush	A, B, C, D, E, F, F*, H, J, M, N, P, Q, S, W, X, Y, Others	Bk, Lv, Rt	0.94	Silva 658	1, 2, 3, 6, 7, 8, 11, 13, 14	*	2.3, 5.3	10
*Bobgunnia madagascariensis* (Desv.) J.H.Kirkbr. & Wiersema	Papilionoideae	Native	BE, BI, CC, CS, CU, HA, HI, LA, LS, LN, MA, NA	Medicinal, Timber, Forage, Poison	A, B, E, F*, H, J, M, Q, T, Others	Bk, Fr, Rt	0.56	Gossweiler 1394	6, 7, 8, 13		5.3	3
*Brachystegia bakeriana* Burtt Davy & Hutch.	Papilionoideae	Native	BI, CC, CS, CU, HA, HI, LS, MO	Medicinal, Timber, Fiber	J, P, R	Bk, St	0.17	Teixeira 32	6, 12, 14		1, 5.3	1
*Brachystegia manga* De Wild.	Papilionoideae	Native	HI	Medicinal	X		0.06	Menezes 1251	6			1
*Brachystegia russelliae* I.M.Johnst.	Papilionoideae	Native	BE, BI, CC, HA, HI, MA	Medicinal, Honey plant	A, Y		0.11	Barbosa & Moreno 12255	6, 14			1
*Brachystegia spiciformis* Benth.	Papilionoideae	Native	BE, BI, BO, CC, CS, CU, HA, HI, LN, LS, MA, MO, NA, UI	Medicinal, Timber, Forage, Fiber, Tanning, Honey plant	B, C, J, R, Others	Rt	0.28	Andrada 53	6, 8, 13, 14		5.3	2
*Brachystegia tamarindoides* Benth.	Papilionoideae	Native	BE, BI, CS, HA, HI, MA	Medicinal, Timber	A, C, F*, H, I, K, Q, U, Y, Others		0.56	Melo & Conceição 42	6, 13		5.3	1
*Burkea africana* Hook.	Caesalpinioideae	Native	BE, BI, CC, CN, CS, CU, HA, HI, LN, LS, MA, MO, NA, UI	Medicinal, Food, Timber, Forage, Ornamental, Honey plant, Tanning, Dye	A, B, C, D, E, G, M, U, Y	Bk, Lv, Rt	0.50	Teixeira 1331	3, 6, 8, 13, 14		5.3	4
*Cassia angolensis* Hiern	Caesalpinioideae	Native	BE, CC, CN, CU, HI, MA, NA	Medicinal, Timber, Ornamental, Shade	B, E, H, J, M, P, Q, T, V, W, X, Y, Others	Bk	0.72	Gossweiler 12840	6, 13, 14		5.3	2
*Cassia psilocarpa* Welw.	Caesalpinioideae	Endemic	MA	Medicinal	E, Q, U, X, Others		0.28	Welwitsch 1740	6		1	1
*Chamaecrista absus* (L.) H.S.Irwin & Barneby	Caesalpinioideae	Native	CC, CN, CS, CU, HI, LA, LN, LS, MA, NA	Medicinal, Forage, Dye	D	Lv	0.06	Torre 108	12, 13, 14		2.3	1
*Chamaecrista biensis* (Steyaert) Lock	Caesalpinioideae	Native	BI, BO, CC, HA, HI, LA	Medicinal, Forage	D, W	Lv	0.11	Antunes vel Dekindt s.n.	3, 13, 14		2.3	2
*Chamaecrista huillensis* (Mendonca & Torre) Lock	Caesalpinioideae	Endemic	CC, CU, HI, NA	Medicinal	C, D, W, Others	Lv	0.22	Teixeira 1934	3, 14		1	3
*Chamaecrista kirkii* (Oliv.) Standl.	Caesalpinioideae	Native	HA, HI, LN, NA, UI	Medicinal, Fiber	B, C, D, E, F*, H, I, J, L, N, T, U, V, X, Others		0.83	Gossweiler 7369	6, 13		5.2	1
*Chamaecrista mimosoides* (L.) Greene	Caesalpinioideae	Native	BE, BO, CC, CN, CS, CU, HA, HI, LA, MA, MO, NA, UI, ZA	Medicinal, Forage, Shade	B, E, V, W	Wp	0.22	Teixeira 944	6, 13, 14		2.3, 5.2	2
*Colophospermum mopane* (Benth.) Leonard	Detarioideae	Native	BE, CU, HI, NA	Medicinal, Timber, Forage, Fiber, Fertilizer, Tanning	B, C, D, E, Q, Y, Others	Lv, Rt	0.39	Teixeira 2542	8, 10, 13	*	5.3	3
*Craibia brevicaudata* subsp. *baptistarum* (Buttner) J.B.Gillett	Papilionoideae	Native	BE, BO, CN, CS, HA, LA, NA, ZA	Medicinal, Forage	D	Lv, Se	0.06	Teixeira 512	10, 14		2.3	1
*Crotalaria abscondita* Baker	Papilionoideae	Native	BI, CC, CN, HA, HI, MA	Medicinal	Y, Z	Wp	0.11	Antunes vel Dekindt s.n.	3, 14			2
*Crotalaria amoena* Baker	Papilionoideae	Native	BE, BI, CC, CN, HA, HI, LN, LS, MO	Medicinal	D, W	Lv, Rt	0.11	Teixeira & Santos 7565	3, 14			3
*Crotalaria anthyllopsis* Baker	Papilionoideae	Native	BE, CS, HA, HI, MA	Medicinal, Food, Fertilizer	D, F, Others	Wp	0.17	Teixeira & Andrade 8316	6, 14		5.2	2
*Crotalaria lachnophora* A.Rich.	Papilionoideae	Native	BE, BI, CC, CN, CS, HA, HI, LS, MA, MO, NA	Medicinal, Food, Forage, Shade	Others		0.06	Antunes vel Dekindt s.n.	13, 14			1
*Crotalaria ononoides* Benth.	Papilionoideae	Native	BE, CC, CN, CS, HA, HI, LS, MA	Medicinal	T, V, X		0.17	Rocha 104	6, 13, 14			2
*Crotalaria pittardiana* Torre	Papilionoideae	Endemic	BE, CC, HA, HI	Medicinal	G, O		0.11	Antunes vel Dekindt 165	14		1	1
*Crotalaria quangensis* Taub.	Papilionoideae	Native	BE, BI, CN, CS, HA, HI, LS, MA, MO	Medicinal	Others		0.06	Gossweiler 12536	2			1
*Crotalaria teixeirae* Torre	Papilionoideae	Native	BE, CC, HI, NA	Medicinal	Others		0.06	Teixeira et al. 12936	6			1
*Cryptosepalum maraviense* Oliv.	Detarioideae	Native	BE, BI, BO, HA, HI, LA, LS, MA	Medicinal	A, Y		0.11	Gossweiler 12515	6, 13			1
*Dalbergia boehmii* Taub.	Papilionoideae	Native	LN	Medicinal, Forage	Q	Rt	0.06	Martins VEG. 66	13, 14			1
*Dalbergia nitidula* Baker	Papilionoideae	Native	BI, CC, CN, CS, CU, HI, MA, MO, NA	Medicinal, Timber	E, F, U, Others	Lv, Rt, St	0.22	Dechamps, Murta & Silva 1553	6, 7, 13		5.3	2
*Desmodium barbatum* (L.) Benth.	Papilionoideae	Native	BE, BI, CC, CN, CS, CU, HA, HI, LN, LS, MA, MO, UI	Medicinal, Forage	D, E	Bk, Lv	0.11	Teixeira 3229	6, 12, 13		2.3	1
*Desmodium hirtum* Guill. & Perr.	Papilionoideae	Native	CN, HI, MA, U	Medicinal, Forage, Cover crop	Others	Rt	0.06	044049 (DR)	11, 13		2.3	1
*Desmodium velutinum* (Willd.) DC.	Papilionoideae	Native	BE, BO, CA, CN, CS, HI, MA, UI, ZA	Medicinal, Forage	I, P	Lv, Rt	0.11	Silva 975	9, 11, 13		2.3	2
*Dialium gossweileri* Baker f.	Dialioideae	Native	CA	Medicinal	I		0.06	Gossweiler 6260	6			1
*Dichrostachys cinerea* (L.) Wight & Arn	Caesalpinioideae	Native	BE, BI, BO, CN, CS, CU, HI, LA, LN, NA, UI, ZA	Medicinal, Food, Timber, Forage, Fiber, Honey plant	A, B, C, D, E, G, H, J, N, P, Q, T, U, W, Z, Others	Bk, Lv, Rt	0.89	Antunes vel Dekindt 909	3, 6, 8, 10, 11, 12, 13, 14	*	2.3, 5.3	7
*Dolichos dongaluta* Baker	Papilionoideae	Endemic	CN, HA, HI, MA, MO	Medicinal	Others	Rt	0.06	Anchieta 78	1, 2, 6		1	3
*Dolichos splendens* Baker	Papilionoideae	Endemic	CN, MA, NA	Medicinal	B, D, N, T, Others		0.28	Antunes vel Dekindt 436	2, 6		1	2
*Droogmansia gossweileri* Torre	Papilionoideae	Endemic	BE, HA	Medicinal	A	Rt	0.06	Gossweiler 10759	8, 13		1	2
*Droogmansia megalantha* (Taub.) De Wild.	Papilionoideae	Native	BE, BI, CC, CS, HA, HI, MA, NA, UI	Medicinal	C, H, U	Rt	0.17	Daniel 11	6, 14			2
*Elephantorrhiza goetzei* (Harms) Harms	Caesalpinioideae	Native	BE, HI, NA	Medicinal, Tanning, Dye	A	Bk, Rt	0.06	Antunes vel Dekindt s.n.	13, 14			2
*Entada abyssinica* A.Rich.	Caesalpinioideae	Native	BE, BI, CN, CS, HA, HI, MA	Medicinal, Timber, Forage, Ornamental, Shade	D, E, F, G, K, N, O, P, Q, T, X, Y, Others	Bk, Br, Fr, Lv, Rt	0.72	Henriques 1049	1, 2, 6, 8, 13		2.3, 5.3	4
*Eriosema affine* De Wild.	Papilionoideae	Native	BI, HA, HI, MA	Medicinal	C, X, Others		0.17	Gossweiler 12195	6			1
*Eriosema albo-griseum* Baker f.	Papilionoideae	Endemic	BE, BI, CS, HA, HI	Medicinal	E	Rt	0.06	Silva 3409	8, 13			1
*Eriosema ellipticum* Baker	Papilionoideae	Native	BI, CC, CS, HA, HI, MA	Medicinal	Q	Rt	0.06	Daniel 12	14			1
*Eriosema glomeratum* (Guill. & Perr.) Hook.f.	Papilionoideae	Native	BO, CA, CN, MO, UI, ZA	Medicinal, Food	B, D, F*, J, U	Lv, Rt, Se	0.28	Gossweiler 10291	3, 9, 13	*		3
*Eriosema griseum* Baker	Papilionoideae	Native	CN, MA, UI	Medicinal	F, V	Lv, Rt	0.11	Gossweiler 5796	11, 13			1
*Eriosema pauciflorum* Klotzsch	Papilionoideae	Native	BI, CC, HI, MA	Medicinal, Forage, Toothbrush	B, Q	Lv, Rt	0.11	Daniel 8	11, 12, 14		1	2
*Eriosema psiloblepharum* Baker f.	Papilionoideae	Endemic	CC, HI, MA	Medicinal	Others		0.06	Antunes vel Dekindt 125	14		1	1
*Erythrina abyssinica* DC.	Papilionoideae	Native	BE, BI, BO, CN, CS, HA, HI, LN, LS, MA, NA, UI	Medicinal, Food, Timber, Forage, Ornamental, Erosion control, Shade, Honey plant, Dye	A, B, D, E, F, G, H, J, K, N, P, Q, T, U, V, X, Z, Others	Bk, Br, Fr, Lv, Rt, Se	1.00	Barbosa & Henriques 9184	6, 7, 8, 9, 11, 13, 14	*	3, 5.2, 5.3	8
*Erythrophleum africanum* (Benth.) Harms	Caesalpinioideae	Native	BE, BI, CC, CN, CS, CU, HA, HI, LN, LS, MA, MO, NA	Medicinal, Timber, Forage, Honey plant, Gum	B, E, F, H, M, N, P, Q, Others	Rt	0.50	Antunes 3146	3, 6, 13, 14		5.3	2
*Erythrophleum letestui* A.Chev.	Caesalpinioideae	Native	CA	Medicinal	Q		0.06	Gossweiler 6132	6			1
*Humularia welwitschii* (Taub.) P.A.Duvign.	Papilionoideae	Native	BE, BI, CN, CU, HA, HI, MA	Medicinal	E, N, X, Z, Others	Rt	0.28	Teixeira 2676	6, 8, 13			2
*Hymenostegia laxiflora* (Benth.) Harms	Detarioideae	Native	BO, CA, CN, CS, LA	Medicinal, gum	C, Q	Rt, Se	0.11	Gossweiler 13950	11, 14			1
*Indigofera antunesiana* Harms	Papilionoideae	Native	BE, BI, CC, CS, CU, HA, HI	Medicinal, Forage	C, E, F*, K, N, Q, V, X, Y		0.50	Teixeira 2766	6, 13			1
*Indigofera charlierana* Schinz	Papilionoideae	Native	CC, CU, HI, NA	Medicinal	D	Wp	0.06	Teixeira & Andrade 8290	3, 12			1
*Indigofera longibarbata* Engl.	Papilionoideae	Native	BE, BI, CC, CN, CS, HA, HI, LS, MA, NA	Medicinal	K		0.06	Santos 464	6			1
*Indigofera spicata* Forssk.	Papilionoideae	Native	BE, CC, CN, LA, MA	Medicinal, Forage, Cover crop, Erosion control	A, C, D, E, W, X		0.33	Teixeira & Andrade 4936	6, 13		2.3,5.2	1
*Indigofera sutherlandioides* Welw. ex Baker	Papilionoideae	Native	BE, BI, CC, HA, HI, LS, MA, MO, NA	Medicinal	G, Others		0.11	Santos 839	6			1
*Isoberlinia angolensis* (Benth.) Hoyle & Brenan	Detarioideae	Native	BE, BI, CN, CS, HA, LS, MA, MO	Medicinal, Timber	D, E, L	Bk, Lv	0.17	Gossweiler 12397	6, 13		5.3	1
*Isoberlinia tomentosa* (Harms) Craib & Stapf	Detarioideae	Native	BI, CS, HA	Medicinal, Food, Timber	G, H, P, Q, T, X		0.33	Dechamps, Murta & Silva 1051	6, 13		5.3	1
*Julbernardia paniculata* (Benth.) Troupin	Detarioideae	Native	BE, BI, CC, CS, CU, HA, HI, LN, LS, MA, MO, NA	Medicinal, Timber, Forage, Honey plant	B, D, E, G, H, J, L, P, Q, R, T, U, X	Bk, Lv	0.72	Andrada 33	6, 7, 8, 13, 14	*	2.3, 5.3	4
*Kotschya strigosa* (Benth.) Dewit & P.A.Duvign.	Papilionoideae	Native	BI, CN, CS, HA, HI, LS	Medicinal	E	Fr	0.06	Teixeira 3394	6, 13			1
*Kotschya strobilantha* (Baker) Dewit & P.A.Duvign.	Papilionoideae	Native	BI, CC, CN, HA, HI, LS, MA, MO	Medicinal	E	Fr	0.06	Gossweiler 3982	6			1
*Lonchocarpus nelsii* (Schinz) Heering & Grimme	Papilionoideae	Native	CC, CU, HI, NA	Medicinal, Timber, Forage	D	Bk	0.06	Teixeira & Andrade 4227	13, 14		2.3, 5.3	1
*Lonchocarpus sericeus* (Poir.) DC.	Papilionoideae	Native	BE, BO, CA, CN, CS, HI, NA, UI	Medicinal, Timber, Ornamental	D, T, X, Others	Bk, Lv, Rt	0.22	Monteiro & Murta 336	1, 6, 13,14		5.3	2
*Millettia aromatica* Dunn	Papilionoideae	Endemic	CN, MA	Medicinal	N, P	St	0.11	Silva 404	2, 6		1	2
*Millettia drastica* Baker	Papilionoideae	Native	BO, CA, CN, CS, LN, MA, UI, ZA,	Medicinal, Ornamental	B	Fr, Se, St	0.06	Monteiro & Murta 69	2, 6, 13			2
*Millettia versicolor* Baker	Papilionoideae	Native	BO, CA, CN, CS, UI, ZA	Medicinal, Timber, Forage, Magic rituals	A, N, Others	Bk, Lv	0.17	Teixeira et al. 11169	9, 11, 13		5.3	2
*Mucuna pruriens* (L.) DC.	Papilionoideae	Native	BE, BO, CA, CN, HA, LS	Medicinal, Food, Forage, Cover crop	B, D, E, F*, H, I, J, N, U, V, W, X, Others		0.72	Exell & Mendonça 378	6, 13		2.3, 5.2	1
*Mucuna stans* Welw. ex Baker	Papilionoideae	Native	BE, BI, CN, HA, HI, MA, NA	Medicinal	B, I, J, K, W	Rt	0.28	Moreno 165	6, 13, 14			2
*Mundulea sericea* (Willd.) A.Chev.	Papilionoideae	Native	CC, CU, HI, NA	Medicinal, Timber, Forage, Poison, Insecticide, Toothbrush	B, I, J, W, Others	Rt	0.28	Gossweiler 2696	6, 13		5.3	1
*Neorautanenia mitis* (A.Rich.) Verdc.	Papilionoideae	Native	BE, BO, CU, HI, LS, MA	Medicinal, Forage	Others	Rt	0.06	Santos & Barroso 2637	13, 14			1
*Peltophorum africanum* Sond.	Caesalpinioideae	Native	BE, CC, CU, HI, NA	Medicinal, Timber, Forage, Honey plant	B, C, K, Y	Bk, Lv	0.22	Santos 258	3, 6, 7, 13, 14		2.3, 5.3	4
*Pentaclethra macrophylla* Benth.	Caesalpinioideae	Native	CA, CN, UI	Medicinal, Food, Timber, Forage, Oil	B, D, Others	Bk, Fr, Lv	0.17	Cameira 119	13	*	2.3, 3, 5.3	1
*Pericopsis angolensis* (Baker) Meeuwen	Papilionoideae	Native	BE, BI, CC, CN, CS, CU, HA, HI, LN, LS, MA, NA	Medicinal, Timber, Forage, Poison, Magic rituals	A, C, H, Q	Bk, Lv, Rt	0.22	Menezes 2421	7, 8, 13		2.3, 5.3	2
*Philenoptera pallescens* (Welw. ex Baker) Schrire	Papilionoideae	Endemic	BE, BO, CN, CS, HI, LA, NA	Medicinal, Honey plant	D, Others	Bk	0.11	Teixeira & Andrade 4227	6, 13, 14			1
*Physostigma mesoponticum* Taub.	Papilionoideae	Native	BE, CU, HA, HI, LS, MA, NA	Medicinal	J, W		0.11	Gossweiler 12184	6, 13			1
*Piptadeniastrum africanum* (Hook.f.) Brenan	Caesalpinioideae	Native	CA, CN, CS, LN	Medicinal, Timber, Forage, Poison, Shade, Fiber, Honey plant	A, E, N	Bk, Rt	0.17	M.E.F.A. 623	6, 13, 14		3, 5.3	1
*Pseudeminia benguellensis* (Torre) Verdc.	Papilionoideae	Endemic	BE, BI, CC, HA, HI	Medicinal	B, E, K, T, U, X, Others		0.39	Gossweiler 125	6		1	1
*Pseudeminia muxiria* (Baker) Verdc.	Papilionoideae	Endemic	CS, MA	Medicinal	B, E, K, T, U, X, Others		0.39	Gossweiler 5992	6		1	1
*Pterocarpus angolensis* DC.	Papilionoideae	Native	BE, BI, CC, CN, CS, CU, HA, HI, LN, LS, MA, MO, NA, UI	Medicinal, Timber, Forage, Dye, Cosmetic, Fiber, Honey plant, Poison	A, B, C, D, E, F, H, P, Q, R, U, V, W, X, Y, Z, Others	Bk, Rt, Sa, St	0.94	Teixeira & Pedro 7599	1, 3, 6, 7, 8, 12, 13, 14	*	1, 2.1, 2.3, 5.3, 12	7
*Pterocarpus lucens* subsp. *antunesii* (Taub.) Rojo	Papilionoideae	Native	BE, BI, CU, HI, NA	Medicinal, Food, Timber, Forage	B, D, V, W, Y	Bk, Fr, Lv, Sa	0.28	Gossweiler 8294	3, 6, 12, 13		2.3, 5.3	2
*Pterocarpus tinctorius* Welw.	Papilionoideae	Native	BO, CN, CS, LA, LN, MA, ZA	Medicinal, Timber, Forage, Dye, Shade	C, Q, U, Others	Bk, Rt, St	0.22	Gossweiler 5915 b	1, 13	*	2.3,3, 5.3	2
*Rhynchosia dekindtii* Harms	Papilionoideae	Endemic	BE, HI	Medicinal	F	Fl, Lv	0.06	Dekindt s.n.	6, 14		1	2
*Rhynchosia insignis* (O.Hoffm.) R.E.Fr.	Papilionoideae	Native	BI, HA, HI, LS, MA	Medicinal	V, Z, Others		0.17	Monteiro & Murta 1609	6, 13			1
*Rhynchosia minima* (L.) DC.	Papilionoideae	Native	BE, BI, BO, CN, CS, CU, HI, LA, MA, NA, UI	Medicinal, Forage	X, Y		0.11	Teixeira 683	6, 12, 13		2.3	1
*Senna singueana* (Delile) Lock	Caesalpinioideae	Native	BE, CS, CU, HI, MO, NA	Medicinal, Food, Timber, Forage, Ornamental, Dye, Tanning	B, C, D, E, Q, U	Fl, Lv, Rt	0.33	Teixeira & Santos 3896	2, 3, 5, 6, 7, 13, 14	*	2.3, 5.3	8
*Sesbania macrantha* Welw. ex E.Phillips & Hutch.	Papilionoideae	Native	BE, BI, CC, HA, HI, LS, MA, NA	Medicinal, Food, Forage, Shade, Soap substitute	G	Lv	0.06	Gossweiler 12546	6, 13		2.3, 5.2	1
*Sesbania pachycarpa* subsp*. pachycarpa* DC.	Papilionoideae	Native	CN, CS, HI, MA, NA	Medicinal, Food, Forage, Fiber	B, D, E, F*, H, I, J, N, T, U, V, W, X, Others		0.78	Teixeira 1338	6, 13	*	2.3, 5.2	2
*Sesbania sesban* (L.) Merr.	Papilionoideae	Native	CU, HA, HI, NA	Medicinal, Food, Timber, Forage, Fiber, gum, Poison	C, G, O	Bk, Lv, Rt	0.17	Teixeira 1603	13	*	2.3, 5.2, 5.3	1
*Sphenostylis stenocarpa* (A.Rich.) Harms	Papilionoideae	Native	BE, CC, CN, LS, MA, MO, UI	Medicinal, Food, Forage	D, N, V, X, Y, Others	Rt	0.33	Machado 292	6, 13	*	3, 5.2	2
*Stylosanthes fruticosa* (Retz.) Alston	Papilionoideae	Native	CU, HI, MA	Medicinal, Forage	D		0.06	Teixeira 1645	13, 14		2.3, 5.2	1
*Tamarindus indica* L.	Detarioideae	Native	CN, HI, LA, NA	Medicinal, Food, Timber, Forage, Honey plant, Dye	B, C, D, E, F, M, Q, V, X, Others	Bk, Fr, Lv, Rt, Sa	0.56	Silva 769	1, 4, 6, 12, 13	*	2.3, 5.3	4
*Tephrosia bracteolata* Guill. & Perr.	Papilionoideae	Native	CN, CS, HI, MA	Medicinal, Forage	B, E, F, Q, T, Others	Lv, Rt	0.33	Teixeira et a. 11434	13	*	3	1
*Tephrosia melanocalyx* Baker	Papilionoideae	Endemic	CC, HI	Medicinal	F	Lv	0.06	Antunes vel Dekindt s.n.	3, 14			2
*Tephrosia vogelii* Hook.f.	Papilionoideae	Native	BI, CA, CN, HA, HI, LN, MA, MO	Medicinal, Forage, Poison, Insecticide, Windbreak, Shade	B, D, M, T, Others	Br, Lv, Rt	0.28	Cardoso s.n	6, 8, 9, 13		5.2	3
*Tylosema fassoglensis* (Schweinf.) Torre & Hillc.	Cercidoideae	Native	BE, BI, CN, CS, CU, HA, HI, LS, MA, UI	Medicinal, Food, Forage, Fiber, Dye	E	Lv	0.06	Teixeira & Andrade 4348	3, 6, 13, 14		5.2	3
*Vigna antunesii* Harms	Papilionoideae	Native	BE, CC, HA, HI, LS, MA, UI	Medicinal, Food	N, Q, W, Others		0.22	Gossweiler 1932	6, 13		5.2	1
*Vigna platyloba* Hiern	Papilionoideae	Native	BI, CS, HA , LN, LS, MA	Medicinal	N, Q, V, Others		0.22	Barbosa 12102	6			1
*Vigna unguiculata* (L.) Walp.	Papilionoideae	Native	BE, BI, BO, CC, CN, CS, CU, HA, HI, LA, MA, NA, ZA	Medicinal, Food, Forage, Fiber	N, Y, Z, Others		0.22	Menezes 2343	7, 13		2.3, 5.2	1
*Vigna vexillata* (L.) A.Rich.	Papilionoideae	Native	BE, BI, CA, CN, HI, LN, LS, NA	Medicinal, Food, Forage, Cover crop, Erosion control	F	Rt	0.06	Matos 43	7, 13	*	2.3, 5.2	2

**Notes.**

a **Main distribution in Angola**: Distribution in Angola. Provinces: BE, Benguela; BI, Bié; BO, Bengo; CA, Cabinda; CC, Cuando-Cubango; CN, Cuanza Norte; CS, Cuanza Sul; CU, Cunene; HA, Huambo; HI, Huíla; LA, Luanda; LN, Lunda Norte; LS, Lunda Sul; MA, Malanje; MO, Moxico; NA, Namibe; UI, Uíge; ZA, Zaire.

b **Main Groups of Diseases**: **A**, pains; **B**, intestinal problems; **C**, stomach problems; **D**, skin inflammations, wounds and burns; **E**, cough, and respiratory diseases; **F**, fever, malaria (species indicated specifically for malaria are with F*); **G**, stings, bites and poisoning; **H**, mental and neurological disorders; **I**, anemia and blood disorders; **J**, diseases of the eyes; **K**, diseases of the liver and gallbladder; **L**, diseases of the kidney; **M**, hearth diseases; **N**, rheumatism and arthritis; **O**, bones and joints; **P**, headaches; **Q**, tooth and mouth diseases; **R**, hair problems; **S**, hemorrhoids; **T**, internal parasites; **U**, sexually transmitted diseases; **V**, infertility; **W**, bladder and urinary problems; **X**, pregnancy, childbirth, breastfeeding and diseases of the new-born; **Y**, menstrual problems and uterine disorders; **Z**, male impotence; **Others** (i.e., other diseases includes sore throat, gout, nasal bleeding, scurvy, leprosy, earache, appetite disorders, obesity, hydrops, coma, rickets, anaesthetic, smallpox, polio, cancer, syncope and paralysis).

c **Parts used in medicine:** **Ae**, aerial parts of plant; **Bk**, bark; **Br**, branches; **Fl**, flowers; **Fr**, fruits; **Lv**, leaves; **Rt**, roots; **Sa**, sap; **Se**, seeds; **St**, stem; **Wp**, whole plant; **Yl**, young leaves.

d **PI:** relative importance of each species indicating the most versatile species (value=1) with the greatest number of medicinal properties.

e **Voucher**: Vouchers are stored in LISC Herbarium, with the exception of *D. hirtum* (in the Herbarium Dresdense).

f **References:** **1**, Ficalho (1947); **2**, Gossweiler (1953); **3**, Peres (1959); **4**, Santos (1967); **5**, Van-Dúnem (1994); **6**, Bossard (1996); **7**, Costa, Dombo & Paula (2009); **8**, Leyens & Lobin (2009); **9**, Göhre et al. (2016); **10**, Bruschi et al. (2017); **11**, Heinze et al. (2017); **12**, IUCN (2018) Red List; **13**, Prota; **14**, Specimens of LISC Herbarium.

g **Healers information**: Species marked with * were mentioned by the healers during author (EC) field surveys.

h **Potential Threats according to IUCN Red List**: **1**, Residential & commercial development; **2**, Agriculture & aquaculture (**2.1** Annual & perennial non-timber crops; **2.3**, Livestock farming & ranching); **3**, Energy production & mining; **5**, Biological resource use (**5.2**, Gathering terrestrial plants; **5.3**, Logging & wood harvesting); **7**, Natural system modifications; **11**, Climate change & severe weather; **12**, Other Options (Source: http://www.iucnredlist.org/technical-documents/classification-schemes/threats-classification-scheme). More details in Data S3.

i **Number of information sources** is the number of different sources that refers to the medicinal uses, including herbarium specimens, bibliographic sources, and fieldwork references.

After compiling the database of the medicinal legumes of Angola, we calculated the pharmacological importance (PI) of each medicinal species by dividing the number of pharmacological properties attributed to the species by the maximum number of properties attributed to the most resourceful species (i.e., the species with the greatest number of pharmacological properties referred). The value of 1 is the highest possible value for PI, indicating the most versatile species with the greatest number of medicinal properties. Pharmacological importance was adapted from  Bennett & Prance (2000) where is designated as “normalized pharmacological properties”. It is a widely used method to measure the species importance in traditional medicine and was previously applied in other studies (e.g., Albuquerque et al., 2007; Giday, Asfaw & Woldu, 2009; Oliveira et al., 2010).

Plant names have been checked and updated according to The Plant List (http://www.theplantlist.org) and the African Plant Database (http://www.ville-ge.ch/musinfo/bd/cjb/africa). The Leguminosae subfamilies were updated following the recent proposed classification of the “Legume Phylogeny Working Group”, which presently recognized six subfamilies: Caesalpinioideae (which also includes the former subfamily Mimosoideae), Cercidoideae, Detarioideae, Dialioideae, Duparquetioideae and Papilionoideae (LPWG, 2017).

## Results

Our results reveal that 127 Leguminosae species and subspecies are recognized as medicinal plants used in Angolan traditional medicine (Table 1). The geographic distribution in Angola, ethnobotanical uses; medicinal applications and plant parts used; other non-medicinal uses (e.g., timber, food and forage); and potential threats for these medicinal plants, are described in Table 1.

The large majority of the species used for medicinal purposes are native non-endemic species (110 species, representing 86.6% of the total), and the endemic plants used as medicinal account for 17 species or 13.4% of the total. The genus *Acacia* have the highest number of medicinal taxa (10), followed by *Crotalaria* with 8 taxa. The most represented Leguminosae subfamilies are Papilionoideae and Caesalpinioideae, with 80 and 35 taxa respectively (Table 1). More than half of the medicinal plants listed in our study are commonly found in three provinces: Huíla (78%), Malanje (54%), and Benguela (54%) while less than 20% is found in Luanda. Overall, Huíla in the South (99 taxa), and Malanje in north-central Angola (69 taxa), boast the greatest diversity of recorded medicinal flora (Table 1; Data S1).

**Figure 1:**
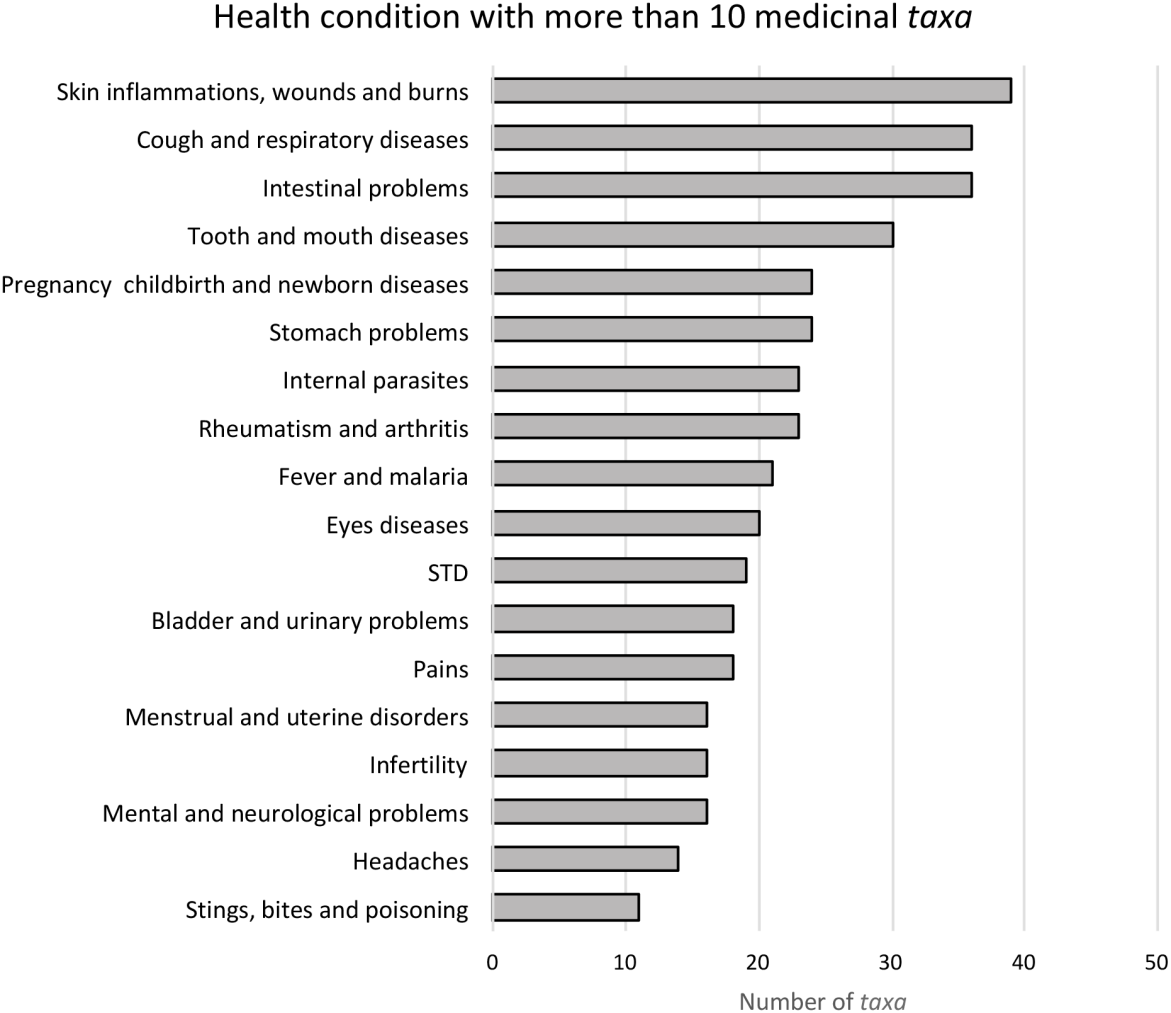
Principal disease groups used by traditional medicine in Angola. Disease groups with fewer than 10 species are not shown (see more details in Table 1).

**Figure 2:**
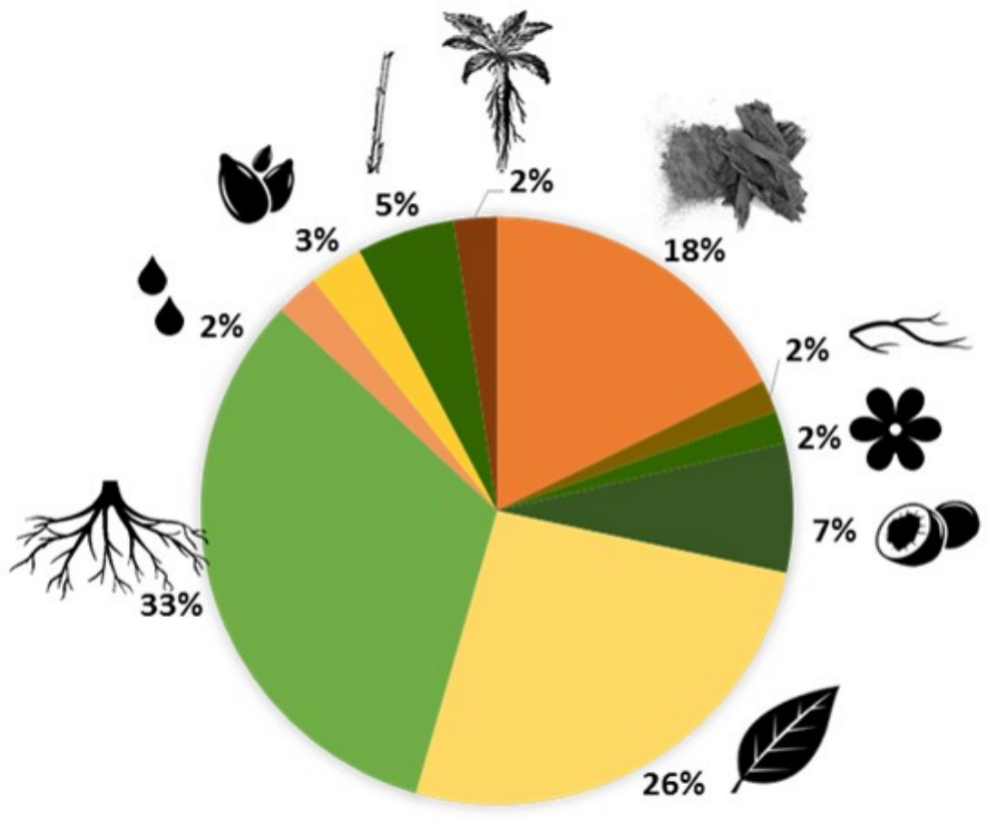
Plant parts used in traditional medicine. Chart: 33%, roots; 26%, leaves; 18%, bark; 7%, fruits; 5%, steam; 3%, seeds; 2%, whole plant; 2%, sap; 2%, flowers; 2%, branches.

**Figure 3:**
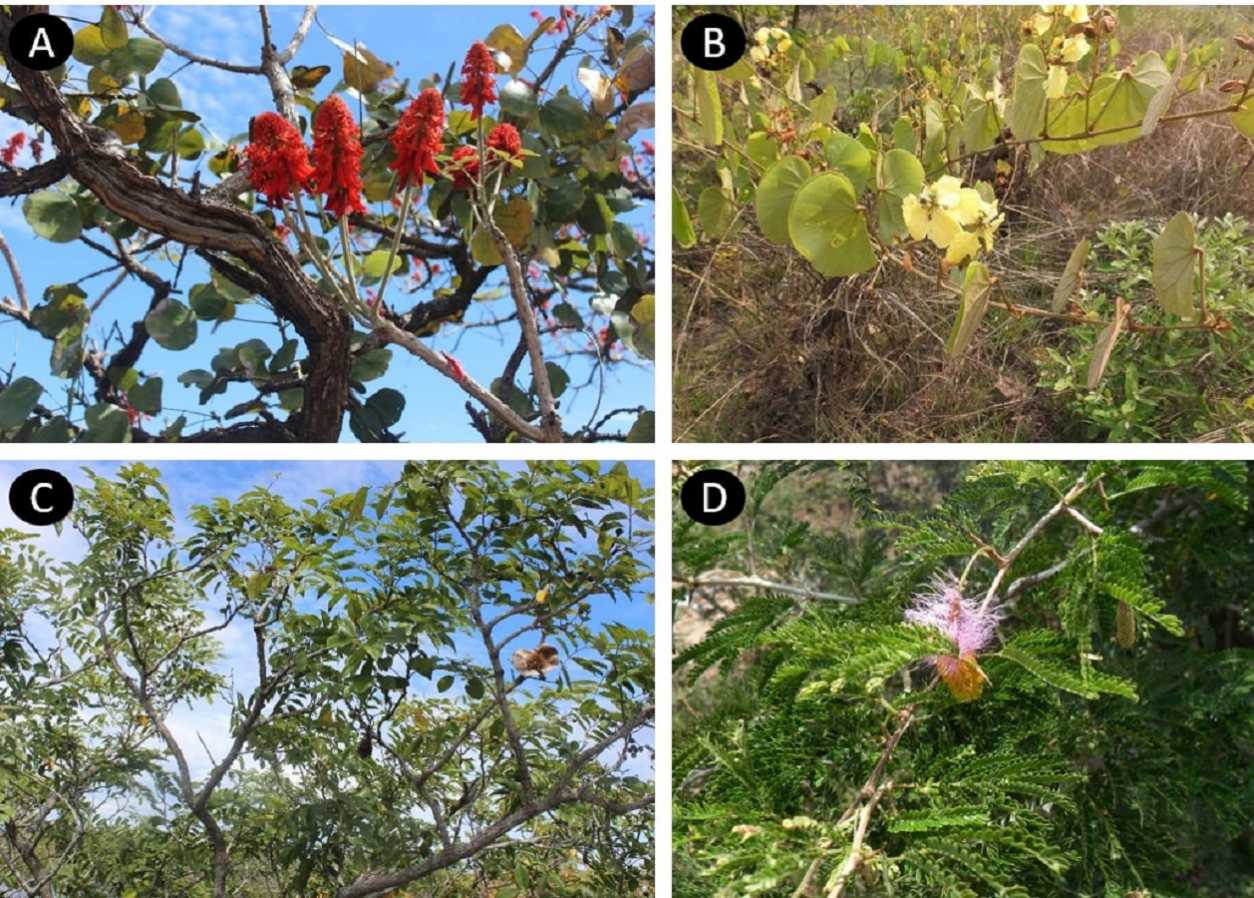
Leguminosae species with more medicinal applications in Angola. *Erythrina abyssinica* (A); *Bauhinia thonningii* (B); *Pterocarpus angolensis* (C); *Dichrostachys cinerea* (D). Photos by Esperança Costa.

The most frequent conditions treated by Leguminosae species are: skin infections, wounds and burns (39 species, 30.7%), intestinal problems (36 species, 28.3%) and respiratory diseases (36 species, 28.3%) (Fig. 1). These plants can be used entirely or only partially, and our study reveals that the most commonly used parts are: roots (33%), leaves (26%) and bark (18%) (Fig. 2). Furthermore, four species of Leguminosae stand out for their high number of medicinal applications: *Erythrina abyssinica* DC. (Fig. 3A) which is used for 18 of the 26 medicinal categories established (PI = 1); the species *Bauhinia thonningii* Schum. (Fig. 3B) and *Pterocarpus angolensis* DC. (Fig. 3C) have 17 applications each (PI = 0.94) followed by *Dichrostachys cinerea* (L.) Wight & Arn (Fig. 3D) with 16 applications (PI = 0.89). Our results also revealed that these species were consistently mentioned as used in traditional medicine by different sources (i.e., herbarium specimens, published sources, and fieldwork) (Table 1).

**Figure 4:**
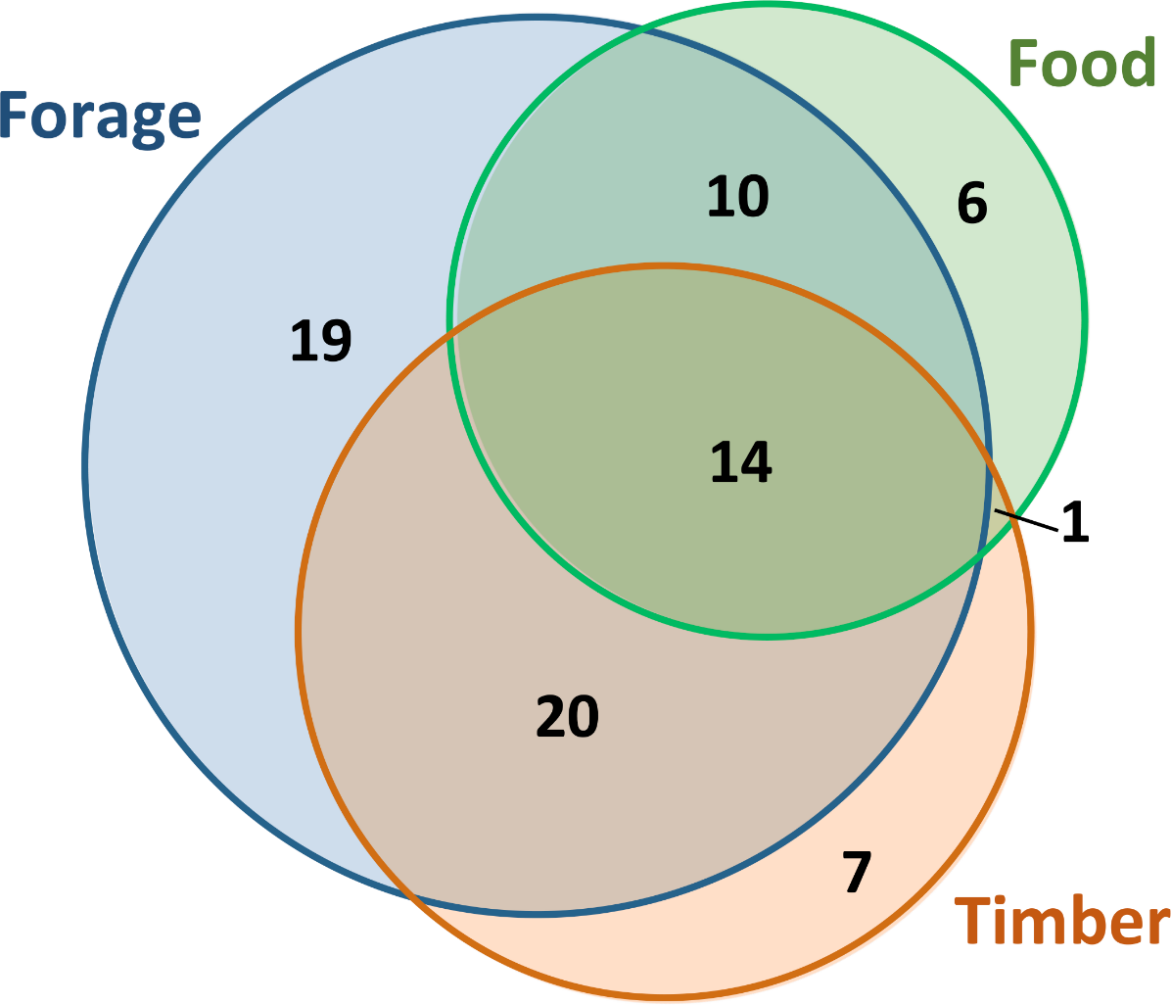
The main uses of medicinal plants in Angola. Euler diagram showing the number of medicinal species with other uses (timber in orange, food in green and forage in blue). The overlapping shapes represent species with two or three uses.

Of the 127 Leguminosae plants used by the local populations, 35% are only used medicinally, while the remaining species were reported to have other uses (Table 1). For instance as forage (63), timber (42), or food (31), and 14 medicinal species overlapping all these uses (Fig. 4). Among these species, some timber trees were identified as particularly vulnerable (e.g., *Acacia goetzei* Harms; *Albizia adianthifolia* (Schum.) W.Wight; *A.  gumifera* (J.F.Gmel.) C.A.Sm.; *Brachystegia spiciformis* Benth.; *B. tamarindoides* Benth.; *Burkea africana* Hook.; *Cassia angolensis* Hiern; *Erythrina abyssinica*; *Julbernardia paniculata* (Benth.) Troupin; *Lonchocarpus sericeus* (Poir.) DC.; *Peltophorum africanum* Sond.; *Piptadeniastrum africanum* (Hook.f.) Brenan; *Pterocarpus angolensis*; *P. tinctorius* Welw.; and *Tamarindus indica* L.) due to the over-collection of their wood in the wild, which was identified as a major threatening factor. Moreover, we identify for each medicinal Leguminosae species the main threats, and our results revealed that 65% of these plants are potentially threatened, mostly as a consequence of: (i) logging & wood harvesting; (ii) increase in livestock farming & ranching; and (iii) gathering terrestrial plants, including harvesting plants, for commercial, subsistence, or cultural purposes (Table 1 and Data S3). Habitat degradation and human disturbance (i.e., residential & commercial development; and natural system modifications) were also reported among the potential threats. Finally, the information available at the IUCN Red List of Threatened Species (IUCN, 2018), revealed that few species were evaluated (12 species, representing 9.4% of the total), among which only *Brachystegia bakeriana* Burtt Davy & Hutch. was classified in a threatened category at global scale, with the Status: Vulnerable B1+2c (Phiri, 1998).

## Discussion

### Leguminosae species used in traditional medicine

The present study is the first survey carried out in Angola to document all the species used in traditional medicine, of one of the biggest plant families - Leguminosae. A total of 127 medicinal plant species were documented, 110 being native non-endemic species, while 17 are endemic species from Angola. Thirty percent of the species have been recorded for the first time as having medicinal uses. Of all the endemic species reported in our study, only three (i.e., *Droogmansia gossweileri* Torre; *Eriosema albo-griseum* Baker f.; and *Philenoptera pallescens* (Welw. ex Baker) Schrire) were reported in current literature about useful plants of tropical Africa (e.g., Burkill, 1994) or in PROTA Web site (http://www.prota.org).

An interesting result of our study was the comparison between our field data and the historical information based on botanical collections. It demonstrated that the Leguminosae medicinal plants recorded several decades ago were also mentioned by the informants during the recent ethnobotanical surveys. Furthermore, it was noted during the field surveys that the knowledge of medicinal plants in Angola is traditionally kept by local healers, but people living in rural areas also demonstrate a broad knowledge of plants and their properties. In general, they maintain a strong relationship with the surrounding environment throughout their lives, using natural resources to supply most of their needs. In fact, our study underlined that some species, which are widely distributed in Angola (e.g., *Bauhinia thonningii*; *Erythrina abyssinica*; and *Pterocarpus angolensis*), are used to treat a relatively large number of illnesses, demonstrating a wide and deep knowledge of medicinal plant properties across the country.

Our findings reveal that 65% of the species reported in our study have their traditional uses confirmed elsewhere in Africa (e.g., Burkill, 1994; PROTA), showing the potential of the Angolan native plants as a source of new compounds of therapeutic interest. The traditional use most frequently noted in our study was the treatment of infectious diseases (e.g., skin infections, intestinal problems and respiratory diseases), which remain the most serious diseases in Africa (Iwu, Duncan & Okunji, 1999). Many of the reported Leguminosae plants have already shown to have interesting biological activity in the treatment of infectious diseases, namely: *Abrus precatorius* L. is traditionally used in Nigeria to treat tuberculosis (Ibekwe et al., 2014); *Albizia adianthifolia* is an important medicinal plant in Guinea-Bissau for alleviating intestinal pain (Catarino, Havik & Romeiras, 2016); and *Pterocarpus angolensis* is used in South Africa for the treatment of parasitic infections affecting humans (Cock, Selesho & Vuuren, 2018).

Since the last decade of the 20th century the prevalence of infectious diseases in Africa increased significantly, and the most important contributing factors are attributed to increases in tuberculosis and HIV/AIDS (Iwu, Duncan & Okunji, 1999). The spread of HIV/AIDS infection has been particularly alarming in sub-Saharan Africa and several studies have focused on plants that can be used in the treatment of this disease (e.g., Mbonu, Borne & Vries, 2009; Becerra, Bildstein & Gach, 2016). Recently, several concerns about the increase of HIV/AIDS were also reported for Angola (e.g., Bártolo et al., 2014) and some species listed in our study (e.g., *Abrus precatorius; Acacia goetzei; Afzelia quanzensis* Welw.; *Dichrostachys cinerea*; *Mucuna pruriens* (L.) DC.; *Peltophorum africanum*; *Sesbania sesban* (L.) Merr.) have shown great promise in the treatment of infectious diseases including opportunistic HIV/AIDS infections (see more details in Chinsembu, 2016). These medicinal plants were highlighted as containing a broad-spectrum of antimicrobial agents used to treat: skin infections, sexually transmitted infections (STI), lung infections such as tuberculosis, pneumonia and cough, and also oral infections, revealing the potential properties of these species for the treatment of HIV/AIDS related diseases.

Similarly, there has been an increased scientific interest in the study of bioactive compounds extracted from plants in prevention and treatment of cancer (Greenwell & Rahman, 2015). For example, *Albizia adianthifolia* extracts collected in Cameroon have revealed considerable cytotoxic activities to fight cancer (Kuete et al., 2016) and ethanol extracts obtained from the roots of *Albizia gummifera* (J.F.Gmel.) C.A.Sm. in Madagascar have shown cytotoxicity against ovarian cancer (Cao et al., 2007). Both species were also reported in our study and the medicinal properties of these species are well-known in Angola (Costa & Pedro, 2013).

With this paper we contribute to update estimations of medicinal plants from Angola and it is highlighted the enormous therapeutic potential of the Leguminosae species in particular. However, the biological activities of these plants are poorly known and require further research in the laboratory, as only few studies (see Pompermaier et al., 2018) have been carried out with Angolan plants to specifically examine the relationships between the activity of the extracts and their medicinal properties.

### Conservation of Leguminosae species in Angola

The preservation of local knowledge together with the conservation of biodiversity are currently regarded as being of major importance in the development of the sub-Saharan Africa (Okigbo, Eme & Ogbogu, 2008). However, there are still important gaps in available data on the conservation status of most of the African medicinal plants. Presently, it is widely accepted that the Red Lists provides the most comprehensive framework to identify and prioritize threatened species (Romeiras et al., 2016a) and several research initiatives were recently conducted across Africa (e.g., for Cameroon by Onana, Cheek & Pollard (2011), for Ethiopia and Eritrea by Vivero, Kelbessa & Demissew (2005), for Cabo Verde by Romeiras et al. (2016b) and for Morocco by Rankou et al. (2015); Lamrani-Alaoui & Hassikou (2018)). The IUCN Red List assessment for the flora of Angola has not yet been conducted, and only a preliminary survey was recently published (Costa, Dombo & Paula, 2009). In fact, assessing the conservation status of medicinal legumes from Angola is a key challenge, because only twelve species were globally evaluated according to the IUCN Red List Categories and Criteria, and only one species (*Brachystegia bakeriana*) was considered Vulnerable (Phiri, 1998). This assessment was published more than 20 years ago and is cleared outdated. This contrasts with some efforts made in other African countries. For example, the Red List of the Flowering Plants of Cameroon classify 815 species under threat categories, being 9% Leguminosae species (Onana, Cheek & Pollard, 2011), while the Red List of Endemic Trees and Shrubs of Ethiopia and Eritrea reports a worst scenario with 18% of the 135 endangered species, from the family Leguminosae (Vivero, Kelbessa & Demissew, 2005). Therefore, particular attention must be given to initiatives for conserving useful plant species, which corresponds to 21% of the threatening processes for vascular plant species assessed on the IUCN Red List (Bachman et al., 2016).

Angola urgently needs to promote actions that will conserve its great diversity of species and also its unique ecosystems, namely: Angolan Miombo woodlands, Angolan Montane Forest-Grassland Mosaic and Angolan scarp savanna, where several medicinal plants thrive. Our results revealed that 38 of the studied taxa are trees, of which 25 are widely used for timber as well as being medicinal. Several of these tree species have a restricted distribution in Angola, namely: (i) *Brachystegia tamarindoides* Benth. has limited distribution in Africa, occurring mainly in Angola in the Miombo area (Exell & Mendonça, 1956); (ii) *Albizia gummifera* and *Pentaclethra macrophylla* Benth. are mainly distributed in the Atlantic Equatorial forests of Cabinda (Exell & Mendonça, 1956); and (iii) *Colophospermum mopane* (Benth.) occurs only in the southern provinces of Angola, mainly in the Mopane woodlands (Makhado et al , 2014; Duvane et al., 2017). On the other hand, *Brachystegia spiciformis, Burkea africana, Julbernardia paniculata* and *Pterocarpus angolensis* are widespread in savannas and Miombo woodlands (Moura et al., 2017). However, several of these tree species are overexploited and are being cut down on a large scale because they are widely used for charcoal production and much valued as timber for furniture and construction, fetching high prices in international markets (Romeiras et al., 2014).

The sustainable management of the legume species is very important as it is crucial to conserve these unique genetic resources. When roots and whole-plants are harvested is clearly more destructive to medicinal plants than collecting their leaves, flowers or fruits (Hamilton, 2004; Kurian & Sankar, 2007). Therefore, vulnerability of species depends on the parts of plants used and how they are collected. For Angola good harvesting practices must be formulated, particularly when collecting roots, which correspond to 33% of the most frequently parts of legume plants used in traditional medicine (see Fig. 2).

For each medicinal species considered in our study, information on other uses was also compiled (i.e., forage; timber; and for human food and livestock feed), which in turn provided further inputs to identify potential threats to the Angola’s medicinal species. The most serious threats identified in our study were logging and wood harvesting and gathering terrestrial plants, which were generally related to over-harvesting, partly due to the collection of plants for other purposes than medicinal. Moreover, habitat degradation and human disturbance, were reported as main threats to species occurring in the surrounding areas of Luanda (the country’s capital and largest city), and in this field the potential threats are highlighted with regard to several endemic species, namely *Acacia welwitschii* Oliv. and *Philenoptera pallescens*, which occur in coastal areas from Luanda to southern parts of Angola.

Moreover, in the scope of this paper, Angola’s medicinal flora has been documented in accordance with their native distribution by the 18 provinces, and notwithstanding notable regional variations, more than half of the medicinal Leguminosae plants are found in southern areas of the country, namely in the regions of Huíla and Benguela, but also in Malanje in north-central Angola. A significant number of species were reported in these 3 provinces to treat a wide range of health conditions, thereby illustrating the need for conserving these species and threatened habitats. Although Angola has established a network of nature reserves and protected areas, encompassing more than 12% of the total land area (MINUA, 2014), there are some limitations in their management structures, namely insufficient financial and staffing commitments. The need for much greater effort and investment in the conservation of threatened species beyond protected areas where most plant diversity occurs, are currently stressed and considerable efforts have been made to identify Important Plant Areas (IPAs) throughout the world (Heywood, 2018). The IPA programme is a mean of identifying and protecting the most important sites for wild plant and habitats, and also offer protection to a wide range of species including medicinal plants, and many common but declining species (Anderson, 2002). IPAs have been delimited for several countries (e.g., De Dios et al., 2017 and specifically to conserve medicinal plants (Hamilton & Radford, 2007). The identification of IPAs in Angola could be based on the presence of threatened plant species under criterion A or based in high concentration of socio-economically important wild-harvested species (including medicinal plants, food plants, timber species, etc.) under sub-criterion B(iii) (Darbyshire et al., 2017). Therefore, our study could provide data towards long term conservation of key sites for plant diversity—IPAs, namely to preserve medicinal species on Huíla, Malanje and Benguela, where the protected area system is not yet representative and comprehensive for safeguarding its botanical diversity. It must be emphasized that in Huíla the existing protected area of Bicuar National Park was historically created to protect big mammals (e.g., black buffalo, antelopes, and elephants), and Cangandala National Park, in Malanje, to protect only one species—the giant sable antelope “Palanca Negra Gigante” (*Hippotragus niger variani*). Therefore, more studies are needed to understand whether medicinal plants are being overexploited within these protected areas in order to suggest conservation strategies for their future preservation.

## Conclusions

This study highlights the importance to proceed with new ethnobotanical studies in developing countries. In particular, the rich plant diversity of Angola suggests a tremendous potential for the discovery of new medicines with considerable therapeutic value. Thus it is essential that further research on the traditional uses of plants by local populations must be carried out, which will require: (i) systematic field surveys, including interviews with local communities; (ii) a review of medicinal data and assembling a specialized literature; and (iii) international cooperation to enable the recovery of scientific knowledge associated with botanical collections, mostly kept in European herbaria. These initiatives will facilitate the undertaking of ethnobotanical studies, particularly where recent field surveys are still lacking, as it happens in most of the Angolan provinces.

Angola is among the sub-Saharan African countries dealing with a crisis of Human Resources for Health (HRH) (Craveiro & Dussault, 2016). Only the populations living in large towns, such as Luanda and other provincial capitals, have access to health facilities and the medicinal plants are still widely used across the country, as they are effective, cheap, used for cultural reasons, and readily available (Macaia & Lapão, 2017). Thus, priority should be given to initiatives for preventing the loss of local knowledge in this country, and for identifying relevant gaps with regard to the conservation and sustainable use of the medicinal plant diversity.

##  Supplemental Information

10.7717/peerj.6736/supp-1 Data S1 Study area with information about field surveys and healers

10.7717/peerj.6736/supp-2 Data S2 Biomes and Ecoregions of Angola (adapted from WWF: http://www.worldwildlife.org)

10.7717/peerj.6736/supp-3 Data S3 Threats Classification Scheme, version 3.2 (adapted from IUCN: http://www.iucnredlist.org)

## Additional Information and Declarations

### Data Availability

The following information was supplied regarding data availability: All the data are available in Table 1.

## References

[ref-1] Albuquerque UP, Monteiro JM, Ramos MA, De Amorim ELC (2007). Medicinal and magic plants from a public market in northeastern Brazil. Journal of Ethnopharmacology.

[ref-2] Albuquerque UP, Ramos MA, De Lucena RFP, Alencar NL, Albuquerque U, Cruz da Cunha L, De Lucena R, Alves R (2014). Methods and techniques used to collect ethnobiological data. Methods and techniques in ethnobiology and ethnoecology, Springer Protocols Handbooks.

[ref-3] Anderson S (2002). Identifying important plant areas.

[ref-4] Bachman S, Fernandez EP, Hargreaves S, Lughadha EN, Rivers M, Williams E, Kew RBG (2016). Extinction risk and threats to plants. State of the world’s plants report—2016.

[ref-5] Bártolo I, Zakovic S, Martin F, Palladino C, Carvalho P, Camacho R, Thamm S, Clemente S, Taveira N (2014). HIV-1 diversity, transmission dynamics and primary drug resistance in Angola. PLOS ONE.

[ref-6] Becerra JC, Bildstein LS, Gach JS (2016). Recent insights into the HIV/AIDS pandemic. Microbial cell.

[ref-7] Bennett BC, Prance GT (2000). Introduced plants in the indigenous pharmacopoeia of Northern South America. Economic Botany.

[ref-8] Bossard E (1996). La medecine traditionnelle au centre et a l’ouest de l’Angola.

[ref-9] Bruschi P, Urso V, Solazzo D, Tonini M, Signorini MA (2017). Traditional knowledge on ethno-veterinary and fodder plants in South Angola: an ethnobotanic field survey in Mopane woodlands in Bibala, Namibe province. Journal of Agriculture and Environment for International Development.

[ref-10] Burgess N, Hales JA, Underwood E, Dinerstein E, Olson D, Itoua I, Schipper J, Ricketts T, Newman K (2004). Terrestrial ecoregions of Africa and Madagascar: a conservation assessment.

[ref-11] Burkill HM (1994). The useful plants of west tropical Africa. 2: Families EI.

[ref-12] Cao S, Norris A, Miller JS, Ratovoson F, Razafitsalama J, Andriantsiferana R, Rasamison VE, TenDykell K, Suhll T, Kingston DG (2007). Cytotoxic Triterpenoid Saponins of Albizia gummifera from the Madagascar Rain Forest 1. Journal of Natural Products.

[ref-13] Catarino L, Havik PJ, Romeiras MM (2016). Medicinal plants of Guinea-Bissau: therapeutic applications, ethnic diversity and knowledge transfer. Journal of Ethnopharmacology.

[ref-14] Chinsembu KC (2016). Ethnobotanical study of plants used in the management of HIV/AIDS-related diseases in Livingstone, Southern Province, Zambia. Evidence-Based Complementary and Alternative Medicine.

[ref-15] Cock IE, Selesho MI, Van Vuuren SF (2018). A review of the traditional use of southern African medicinal plants for the treatment of selected parasite infections affecting humans. Journal of Ethnopharmacology.

[ref-16] Costa E, Dombo A, Paula M (2009). Plantas ameacadas em Angola̧.

[ref-17] Costa E, Pedro M (2013). Plantas medicinais de Angola.

[ref-18] Craveiro I, Dussault G (2016). The impact of global health initiatives on the health system in Angola. Global Public Health.

[ref-19] Darbyshire I, Anderson S, Asatryan A, Byfield A, Cheek M, Clubbe C, Ghrabi Z, Harris T, Heatubun CD, Kalema J, Magassouba S, McCarthy B, Milliken W, De Montmollin B, Lughadha EN, Onana JM, Saïdou D, Sârbu A, Shrestha K, Radford E (2017). Important plant areas: revised selection criteria for a global approach to plant conservation. Biodiversity and Conservation.

[ref-20] De Dios RS, Ruano CC, Lozano FD, Ollero HS, Saiz JC (2017). The role of criteria in selecting important areas for conservation in biodiversity-rich territories. Diversity and Distributions.

[ref-21] Duvane JA, Jorge TF, Maquia I, Ribeiro N, Ribeiro-Barros AI, António C (2017). Characterization of the Primary Metabolome of *Brachystegia boehmii* and Colophospermum mopane under Different Fire Regimes in Miombo and Mopane African Woodlands. Frontiers in Plant Science.

[ref-22] Exell AW, Fernandes A (1962). Conspectus Florae Angolensis III, Fasc. 1. Leguminosae (Papilionoideae: Genisteae-Galegeae.

[ref-23] Exell AW, Fernandes A (1966). Conspectus Florae Angolensis III, Fasc. 2. Leguminosae (Papilionoideae: Hedysareae-Sophoreae).

[ref-24] Exell AW, Mendonça FA (1956). Conspectus Florae Angolensis II, Fasc. 2. Leguminosae (Caesalpinioideae-Mimosoideae).

[ref-25] Ficalho C (1947). Plantas úteis da África portuguesa.

[ref-26] Figueiredo E, Smith GF (2008). Plants of angola/plantas de angola.

[ref-27] Giday M, Asfaw Z, Woldu Z (2009). Medicinal plants of the Meinit ethnic group of Ethiopia: an ethnobotanical study. Journal of Ethnopharmacology.

[ref-28] Göhre A, Toto-Nienguesse ÁB, Futuro M, Neinhuis C, Lautenschläger T (2016). Plants from disturbed savannah vegetation and their usage by Bakongo tribes in Uíge, Northern Angola. Journal of Ethnobiology and Ethnomedicine.

[ref-29] Gosoniu L, Veta AM, Vounatsou (2010). Bayesian geostatistical modeling of malaria indicator survey data in Angola. PLOS ONE.

[ref-30] Gossweiler J (1953). Nomes indigenas de plantas de Angola.

[ref-31] Graham PH, Vance CP (2003). Legumes: importance and constraints to greater use. Plant Physiology.

[ref-32] Greenwell M, Rahman PKSM (2015). Medicinal plants: their use in anticancer treatment. International Journal of Pharmaceutical Sciences and Research.

[ref-33] Hamilton AC (2004). Medicinal plants, conservation and livelihoods. Biodiversity & Conservation.

[ref-34] Hamilton AC, Radford EA (2007). Identification and conservation of Important Plant Areas for medicinal plants in the Himalaya. https://www.plantlife.org.uk/uk/our-work/publications/identification-and-conservation-important-plant-areas-medicinal-plants-himalaya.

[ref-35] Hansen MC, Potapov PV, Moore R, Hancher M, Turubanova SAA, Tyukavina A, Thau D, Stehman SV, Goetz SJ, Loveland TR, Kommareddy A, Egorov A, Chini1 L, Justice CO, Townshend JRG (2013). High-resolution global maps of 21st-century forest cover change. Science.

[ref-36] Heinze C, Ditsch B, Congo MF, José IJ, Neinhuis C, Lautenschlaeger T (2017). First Ethnobotanical Analysis of Useful Plants in Cuanza Norte, North Angola. Research & R eviews: Journal of Botanical Sciences.

[ref-37] Heywood VH (2018). Conserving plants within and beyond protected areas–still problematic and future uncertain. Plant Diversity.

[ref-38] Howieson JG, Yates RJ, Foster KJ, Real D, Besier RB, Dilworth MJ, James EK, Sprent JI, Newton WE (2008). Prospects for the future use of legumes. Leguminous nitrogen-fixing symbioses.

[ref-39] Ibekwe NN, Nvau JB, Oladosu PO, Usman AM, Ibrahim K, Boshoff HI, Dowd CS, Orisadipe AT, Aiyelaagbe O, Adesomoju AA, Barry IIICE (2014). Some Nigerian anti-tuberculosis ethnomedicines: a preliminary efficacy assessment. Journal of Ethnopharmacology.

[ref-40] Instituto Nacional de Estatística (INE) (2014). Resultados Preliminares do Recenseamento Geral da Populacão e da Habitação de Angola 2014.

[ref-41] IUCN (2018). http://www.iucnredlist.org.

[ref-42] Iwu MW, Duncan AR, Okunji CO, Janick J (1999). New antimicrobials of plant origin. Perspectives on new crops and new uses.

[ref-43] Kuete V, Sandjo LP, Wiench B, Efferth T (2013). Cytotoxicity and modes of action of four Cameroonian dietary spices ethno-medically used to treat Cancers: *Echinops giganteus*, *Xylopia aethiopica*, *Imperata cylindrica* and *Piper capense*. Journal of Ethnopharmacology.

[ref-44] Kuete V, Tchinda CF, Mambe FT, Beng VP, Efferth T (2016). Cytotoxicity of methanol extracts of 10 Cameroonian medicinal plants towards multi-factorial drug-resistant cancer cell lines. BMC Complementary and Alternative Medicine.

[ref-45] Kurian A, Sankar MA, Peter KV (2007). Medicinal plants.

[ref-46] Lamrani-Alaoui M, Hassikou R (2018). Rapid risk assessment to harvesting of wild medicinal and aromatic plant species in Morocco for conservation and sustainable management purposes. Biodiversity and Conservation.

[ref-47] Leyens T, Lobin W (2009). Manual de plantas úteis de Angola.

[ref-48] LPWG L (2017). A new subfamily classification of the Leguminosae based on a taxonomically comprehensive phylogeny. The Legume Phylogeny Working Group (LPWG). Taxon.

[ref-49] Macaia D, Lapão LV (2017). The current situation of human resources for health in the province of Cabinda in Angola: is it a limitation to provide universal access to healthcare?. Human Resources for Health.

[ref-50] Mahomoodally MF (2013). Traditional medicines in Africa: an appraisal of ten potent African medicinal plants. Evidence-Based Complementary and Alternative Medicine.

[ref-51] Makhado RA, Mapaure I, Potgieter MJ, Luus-Powell1 WJ, Saidi AT (2014). Factors influencing the adaptation and distribution of *Colophospermum mopane* in southern Africa’s mopane savannas—a review. Bothalia.

[ref-52] Máthé Á, Neffati M, Najjaa H (2017). Medicinal and aromatic plants of the World-Africa, Volume 3.

[ref-53] Mbonu NC, Van den Borne B, De Vries NK (2009). Stigma of people with HIV/AIDS in Sub-Saharan Africa: a literature review. Journal of Tropical Medicine.

[ref-54] Mertz O, Ravnborg HM, Lövei GL, Nielsen I, Konijnendijk CC (2007). Ecosystem services and biodiversity in developing countries. Biodiversity and Conservation.

[ref-55] Millennium Ecosystem Assessment (2005). Millennium ecosystem assessment.

[ref-56] MINUA (Ministry of Urban Affairs and Environment) (2014). 5th National Report on Biodiversity in Angola, 2007–2012.

[ref-57] Moura I, Maquia I, Rija AA, Ribeiro N, Ribeiro-Barros AI, Bitz L (2017). Biodiversity studies in key species from the African Mopane and Miombo Woodlands. Genetic Diversity.

[ref-58] Moyo M, Aremu AO, Van Staden J (2015). Medicinal plants: an invaluable, dwindling resource in sub-Saharan Africa. Journal of Ethnopharmacology.

[ref-59] Najam A, Cleveland CJ (2005). Energy and sustainable development at global environmental summits: an evolving agenda. Environment, Development and Sustainability.

[ref-60] Okigbo RN, Eme UE, Ogbogu S (2008). Biodiversity and conservation of medicinal and aromatic plants in Africa. Biotechnology and Molecular Biology Reviews.

[ref-61] Oliveira ES, Torres DF, Brooks SE, Alves RR (2010). The medicinal animal markets in the metropolitan region of Natal City Northeastern Brazil. Journal of Ethnopharmacology.

[ref-62] Olson DM, Dinerstein E, Wikramanayake ED, Burgess ND, Powell GV, Underwood EC, D’amico JA, Itoua I, Strand HE, Morrison JC, Loucks CJ, Allnutt TF, Ricketts TH, Kura Y, Lamoreux JF, Wettengel WW, Hedao P, Kassem KR (2001). Terrestrial ecoregions of the world: a new map of life on earth: a new global map of terrestrial ecoregions provides an innovative tool for conserving biodiversity. BioScience.

[ref-63] Onana JM, Cheek M, Pollard BJ (2011). Red data book of the flowering plants of Cameroon.

[ref-64] Peres AC (1959). Subsidios para o estudo etno-botânico da Huíla: trabalho realizado no Centro de Botânica da Junta de Investigações do Ultramar.

[ref-65] Phiri PSM (1998). *Brachystegia bakeriana*. The IUCN Red List of Threatened Species 1998: e.T35367A9924043. http://dx.doi.org/10.2305/IUCN.UK.1998.RLTS.T35367A9924043.en.

[ref-66] Pimm SL, Joppa LN (2015). How many plant species are there, where are they, and at what rate are they going extinct?. Annals of the Missouri Botanical Garden.

[ref-67] Pompermaier L, Marzocco S, Adesso S, Monizi M, Schwaiger S, Neinhuis C, Stuppnera H, Lautenschläger T (2018). Medicinal plants of northern Angola and their anti-inflammatory properties. Journal of Ethnopharmacology.

[ref-68] Rankou H, Culham A, Taleb MS, Ouhammou A, Martin G, Jury SL (2015). Conservation assessments and Red Listing of the endemic Moroccan flora (monocotyledons). Botanical Journal of the Linnean Society.

[ref-69] Romeiras MM, Catarino S, Filipe A, Magalhães M, Duarte MC, Beja P (2016a). Species conservation assessments in small islands: the consequences of precautionary versus evidentiary attitudes. Conservation Letters.

[ref-70] Romeiras MM, Catarino S, Gomes I, Fernandes C, Costa JC, Caujapé-Castells J, Duarte MC (2016b). IUCN Red List assessment of the Cape Verde endemic flora: towards a global strategy for plant conservation in Macaronesia. Botanical Journal of the Linnean Society.

[ref-71] Romeiras MM, Figueira R, Duarte MC, Beja P, Darbyshire I (2014). Documenting biogeographical patterns of African timber species using herbarium records: a conservation perspective based on native trees from Angola. PLOS ONE.

[ref-72] Santos RM (1967). Plantas uteis de Angola: contribuição iconográfica I.

[ref-73] Santos RM (1989). Plantas úteis de Angola: contribuição iconográfica II.

[ref-74] Schubert J (2017). Working the system: a political ethnography of the new Angola.

[ref-75] Shamsi TN, Parveen R, Ahamad S, Fatima S (2017). Structural and biophysical characterization of *Cajanus cajan* protease inhibitor. Journal of Natural Science, Biology, and Medicine.

[ref-76] Soares M, Abreu J, Nunes H, Silveira P, Schrire B, Figueiredo E (2007). The Leguminosae of Angola: diversity and endemism. Systematics and Geography of Plants.

[ref-77] Thorn J, Snaddon J, Waldron A, Kok K, Zhou W, Bhagwat S, Willis K, Petrokofsky G (2015). How effective are on-farm conservation land management strategies for preserving ecosystem services in developing countries? A systematic map protocol. Environmental Evidence.

[ref-78] Urso V, Signorini MA, Bruschi P (2013). Survey of the ethnobotanical uses of *Ximenia americana* L.(mumpeke) among rural communities in South Angola. Journal of Medicinal Plants Research.

[ref-79] Van-Dúnem M (1994). Medicamentos ao alcance de todos: plantas medicinais de Angola.

[ref-80] Van Wyk BE (2008). A broad review of commercially important southern African medicinal plants. Journal of Ethnopharmacology.

[ref-81] Vivero JL, Kelbessa E, Demissew S (2005). The red list of endemic trees & shrubs of Ethiopia and Eritrea.

