# Supplementary material for: Conservation and sustainable use of the medicinal Leguminosae plants from Angola
Source: PeerJ. 2019 May 23;7:e6736. doi: 10.7717/peerj.6736 (PMC6535223; doi:10.7717/peerj.6736)
Supplement: Data S1 [file peerj-07-6736-s001.docx]

**
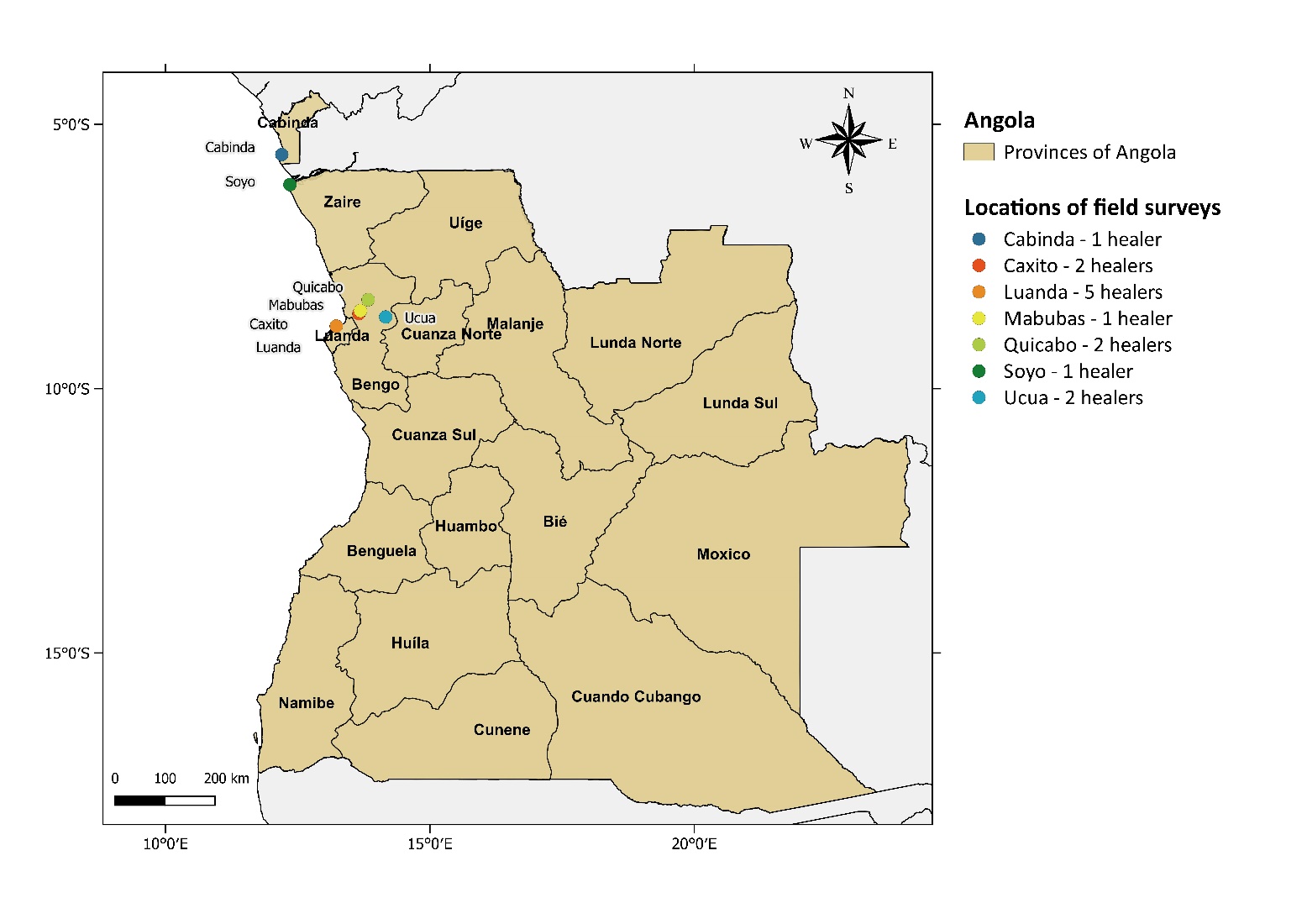
 Supplemental Data S1. Study area with information about field surveys and healers**

| **Healer Name** | **Gender** | **Age** | **Province** | **Location** | **Year** |
| --- | --- | --- | --- | --- | --- |
| Amélia Mugunga | Female | 71 | Bengo | Mabubas | 2008 |
| Antónia Augusto | Female | 82 | Bengo | Caxito | 2011 |
| Carlota Pontes | Female | 65 | Bengo | Quicabo | 2008 |
| Daniel Samuel | Male | 41 | Zaire | Soyo | 2013 |
| Delfina Capita | Female | 83 | Luanda | Luanda | 2009 |
| Eva Afonso | Female | 101 | Bengo | Ucua | 2011 |
| Joana Vieira | Female | 63 | Luanda | Luanda | 2012 |
| Manuel de Carvalho | Male | 61 | Luanda | Luanda | 2013 |
| Maria Mateus | Female | 68 | Luanda | Luanda | 2012 |
| Nphumu Nkonde | Male | 72 | Cabinda | Cabinda | 2013 |
| Quissanga João | Male | 101 | Bengo | Ucua | 2008 |
| Sabina Gomes | Female | 85 | Bengo | Caxito | 2010 |
| Teresa Teixeira | Female | 50 | Luanda | Luanda | 2013 |
| Teresa Tomense | Female | 75 | Bengo | Quicabo | 2009 |

**Healers’ information**:
