# Supplementary material for: Conservation and sustainable use of the medicinal Leguminosae plants from Angola
Source: PeerJ. 2019 May 23;7:e6736. doi: 10.7717/peerj.6736 (PMC6535223; doi:10.7717/peerj.6736)
Supplement: Data S2 [file peerj-07-6736-s002.docx]

**Supplemental Data S2. Biomes and Ecoregions of Angola**


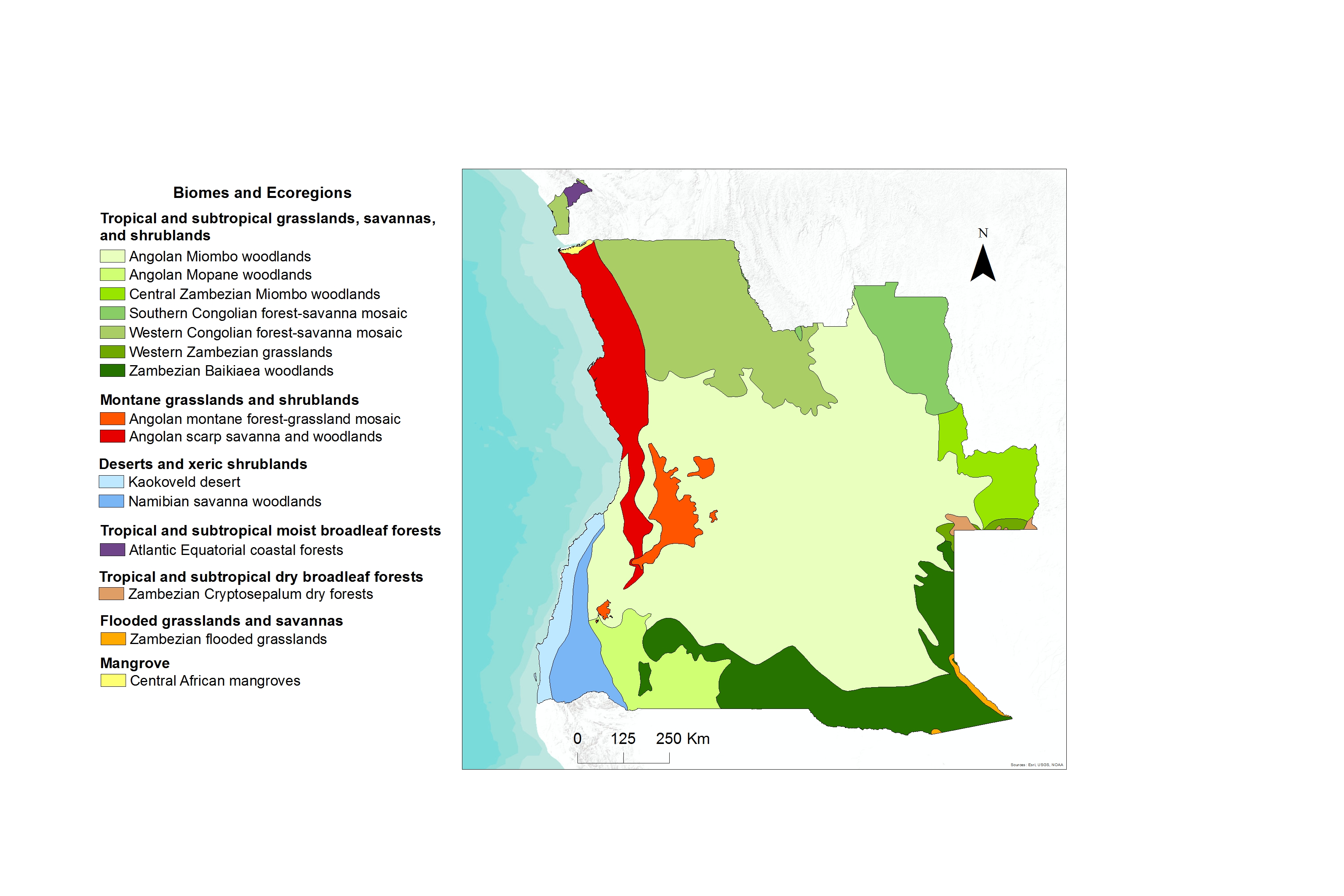


Angola includes 15 ecoregions (see for more details: Olson et al., 2001), which reveals the great variety of climatic and geographic characteristics of this country. These ecoregions are included within seven main biomes:

**1)** **Tropical and subtropical grasslands, savannas, and shrublands**: this is the largest biome that includes seven ecoregions (above map, green areas). Angolan Miombo woodlands occupies the largest area, with species of middle and high altitude, it is a region of poor-soils and low organic matter, where legume species as *Brachystegia spiciformis* and *Julbernardia paniculata* have a very important role fixing nitrogen that improves soil quality (Burgess et al., 2004; Costa & Pedro, 2013). According to World Wildlife Fund (WWF) (Olson et al., 2001) this ecoregion was classified as Vulnerable, and is threatened by habitat fragmentation, hunting and tree cutting for timber, firewood and charcoal manufacture.

**2) Montane grasslands and shrublands biome**: this biome comprise two ecoregions (red areas), Angolan scarp savanna and woodlands; and Angolan montane forest-grassland mosaic, which was classified as Endangered (Olson et al., 2001). This is a complex area with a high diversity of vegetation types and significant levels of endemism. The main threats are: agriculture, woodcutting, harvesting of forest products and livestock grazing, mainly around the larger urban centers (Burgess et al., 2004).

**3) Deserts and xeric shrublands**: composed by two ecoregions (blue areas), this is an important center of Leguminosae endemism. The main threats are soil erosion and loss of grass species diversity.

**4) Tropical and subtropical moist broadleaf forests**: is only represented by the Atlantic Equatorial coastal forest ecoregion (dark violet area), which is restricted to Cabinda enclave. It is a humid and dense forest region with a very rich species diversity, including the emblematic trees, such as the medicinal: *Pterocarpus tinctorius*, *Erythrina abyssinica*, and *Eriosema glomeratum* (Costa & Pedro, 2013; Olson et al., 2001). It is mainly threatened by logging but the forest is considered Relatively Stable (Burgess et al., 2004).

**5) Tropical and subtropical dry broadleaf forests:** this region, including a single ecoregion (brown areas), is poorly studied and the lack of water and the poor soils has resulted in a low population density (INE, 2014). Few threats are known and the region is classified as Relatively Stable (Burgess et al., 2004).

**6) Flooded grasslands and savannas**: comprises the Zambezian flooded grasslands ecoregion (orange areas). In Angola this is a small area, rounding Okavango River, and is integrated in the national protected areas network.

**7)** **Mangrove**: includes the Central African mangroves ecoregion (yellow area). This is a very restricted area near the river Congo, which was classified as Endangered. The main threats are: logging, petroleum exploitation, urbanization, industrialization, and agriculture (Burgess et al., 2004).
