# Supplementary material for: Conservation and sustainable use of the medicinal Leguminosae plants from Angola
Source: PeerJ. 2019 May 23;7:e6736. doi: 10.7717/peerj.6736 (PMC6535223; doi:10.7717/peerj.6736)
Supplement: Data S3 [file peerj-07-6736-s003.docx]

**Supplemental Data S3. Threats Classification Scheme (Version 3.2)**

Classification scheme proposed by IUCN Red List (http://www.iucnredlist.org/technical-documents/classification-schemes/threats-classification-scheme) to identify the potential threats for each species.

| **Potential Threats – IUCN Red List** | | |
| --- | --- | --- |
| 1. **Residential & Commercial Development**   Threats from human settlements or other non-agricultural land uses with a substantial footprint. | | |
|  | 1.1 Housing & Urban Areas | |
|  | 1.2 Commercial & Industrial Areas | |
|  | 1.3 Tourism & Recreation Areas | |
| 1. **Agriculture & Aquaculture**   Threats from farming and ranching as a result of agricultural expansion and intensification, including silviculture, mariculture and aquaculture. | | |
|  | 2.1 Annual & Perennial Non-Timber Crops | |
|  | 2.1.1 Shifting Agriculture | |
|  | 2.1.2 Small-holder Farming | |
|  | 2.1.3 Agro-industry Farming | |
|  | 2.1.4 Scale Unknown/Unrecorded | |
|  | 2.2 Wood & Pulp Plantations | |
|  | 2.2.1 Small-holder Plantations | |
|  | 2.2.2 Agro-industry Plantations | |
|  | 2.2.3 Scale Unknown/Unrecorded | |
|  | 2.3 Livestock Farming & Ranching | |
|  | 2.3.1 Nomadic Grazing | |
|  | 2.3.2 Small-holder Grazing, Ranching or Farming | |
|  | 2.3.3 Agro-industry Grazing, Ranching or Farming | |
|  | 2.3.4 Scale Unknown/Unrecorded | |
|  | 2.4 Marine & Freshwater Aquaculture | |
|  | 2.4.1 Subsistence/Artisinal Aquaculture | |
|  | 2.4.2 Industrial Aquaculture | |
|  | 2.4.3 Scale Unknown/Unrecorded | |
| 1. **Energy Production & Mining**   Threats from production of non-biological resources. | | |
|  | 3.1 Oil & Gas Drilling | |
|  | 3.2 Mining & Quarrying |  |
|  | 3.3 Renewable Energy | |
|  | 1. **Transportation & Service Corridors**   Threats from long narrow transport corridors and the vehicles that use them including associated wildlife mortality. | |
|  | 4.1 Roads & Railroads | |
|  | 4.2 Utility & Service Lines |  |
|  | 4.3 Shipping Lanes | |
|  | 4.4 Flight Paths |  |

| 1. **Biological Resource Use**   Threats from consumptive use of "wild" biological resources including both deliberate and unintentional harvesting effects; also persecution or control of specific species. | |
| --- | --- |
|  | 5.1 Hunting & Collecting Terrestrial Animals |
|  | 5.1.1 Intentional Use (species being assessed is the target) |
|  | 5.1.2 Unintentional effects (species being assessed is not the target) |
|  | 5.1.3 Persecution/Control |
|  | 5.1.4 Motivation Unknown/Unrecorded |
|  | 5.2 Gathering Terrestrial Plants |
|  | 5.2.1 Intentional Use (species being assessed is the target) |
|  | 5.2.2 Unintentional effects (species being assessed is not the target) |
|  | 5.2.3 Persecution/Control |
|  | 5.3 Logging & Wood Harvesting |
|  | 5.3.1 Intentional Use: subsistence/small scale (species being assessed is the target) |
|  | 5.3.2 Intentional Use: large scale (species being assessed is the target) |
|  | 5.3.3 Unintentional effects: subsistence/small scale (species being assessed is not the target) |
|  | 5.3.4 Unintentional effects: large scale (species being assessed is not the target) |
|  | 5.3.5 Motivation Unknown/Unrecorded |
|  | 5.4 Fishing & Harvesting Aquatic Resources |
|  | 5.4.1 Intentional Use: subsistence/small scale (species being assessed is the target) |
|  | 5.4.2 Intentional Use: large scale (species being assessed is the target) |
|  | 5.4.3 Unintentional effects: subsistence/small scale (species being assessed is not the target) |
|  | 5.4.4 Unintentional effects: large scale (species being assessed is not the target) |
|  | 5.4.5 Persecution/Control |
|  | 5.4.6 Motivation Unknown/Unrecorded |
| 1. **Human Intrusions & Disturbance**   Threats from human activities that alter, destroy and disturb habitats and species associated with non-consumptive uses of biological resources. | |
|  | 6.1 Recreational Activities |
|  | 6.2 War, Civil Unrest & Military Exercises |
|  | 6.3 Work & Other Activities |
| 1. **Natural System Modifications**   Threats from actions that convert or degrade habitat in service of “managing” natural or semi-natural systems, often to improve human welfare. | |
|  | 7.1 Fire & Fire Suppression |
|  | 7.1.1 Increase in Fire Frequency/Intensity |
|  | 7.1.2 Suppression in Fire Frequency/Intensity |
|  | 7.1.3 Trend Unknown/Unrecorded |
|  | 7.2 Dams & Water Management/Use |
|  | 7.2.1 Abstraction of Surface Water (domestic use) |
|  | 7.2.2 Abstraction of Surface Water (commercial use) |
|  | 7.2.3 Abstraction of Surface Water (agricultural use) |
|  | 7.2.4 Abstraction of Surface Water (unknown use) |
|  | 7.2.5 Abstraction of Ground Water (domestic use) |
|  | 7.2.6 Abstraction of Ground Water (commercial use) |
|  | 7.2.7 Abstraction of Ground Water (agricultural use) |
|  | 7.2.8 Abstraction of Ground Water (unknown use) |
|  | 7.2.9 Small Dams |
|  | 7.2.10 Large Dams |
|  | 7.2.11 Dams (size unknown) |
|  | 7.3 Other Ecosystem Modifications |
|  | 1. **Invasive & Other Problematic Species, Genes & Diseases**   Threats from non-native and native plants, animals, pathogens/microbes, or genetic materials that have or are predicted to have harmful effects on biodiversity following their introduction, spread and/or increase in abundance. |
|  | 8.1 Invasive Non-Native/Alien Species/Diseases |
|  | 8.1.1 Unspecified Species |
|  | 8.1.2 Named Species |
|  | 8.2 Problematic Native Species/Diseases |
|  | 8.2.1 Unspecified Species |
|  | 8.2.2 Named Species |
|  | 8.3 Introduced Genetic Material |
|  | 8.4 Problematic Species/Diseases of Unknown Origin |
|  | 8.4.1 Unspecified Species |
|  | 8.4.2 Named Species |
|  | 8.5 Viral/Prion-induced Diseases |
|  | 8.5.1 Unspecified "Species" (Disease) |
|  | 8.5.2 Named "Species" (Disease) |
|  | 8.6 Diseases of Unknown Cause |
|  | 1. **Pollution**   Threats from introduction of exotic and/or excess materials or energy from point and nonpoint sources. |
|  | 9.1 Domestic & Urban Waste Water |
|  | 9.1.1 Sewage |
|  | 9.1.2 Run-off |
|  | 9.1.3 Type Unknown/Unrecorded |
|  | 9.2 Industrial & Military Effluents |
|  | 9.2.1 Oil Spills |
|  | 9.2.2 Seepage from Mining |
|  | 9.2.3 Type Unknown/Unrecorded |
|  | 9.3 Agricultural & Forestry Effluents |
|  | 9.3.1 Nutrient Loads |
|  | 9.3.2 Soil Erosion, Sedimentation |
|  | 9.3.3 Herbicides and Pesticides |
|  | 9.3.4 Type Unknown/Unrecorded |
|  | 9.4 Garbage & Solid Waste |
|  | 9.5 Air-Borne Pollutants |
|  | 9.5.1 Acid rain |
|  | 9.5.2 Smog |
|  | 9.5.3 Ozone |
|  | 9.5.4 Type Unknown/Unrecorded |
|  | 9.6 Excess Energy |
|  | 9.6.1 Light Pollution |
|  | 9.6.2 Thermal Pollution |
|  | 9.6.3 Noise Pollution |
|  | 9.6.4 Type Unknown/Unrecorded |
| 1. **Geological Events**   Threats from catastrophic geological events. | |
|  | 10.1 Volcanoes |
|  | 10.2 Earthquakes/Tsunamis |
|  | 10.3 Avalanches/Landslides |

|  | 1. **Climate Change & Severe Weather**   Threats from long-term climatic changes which may be linked to global warming and other severe climatic/weather events that are outside of the natural range of variation, or potentially can wipe out a vulnerable species or habitat. |
| --- | --- |
|  | 11.1 Habitat Shifting & Alteration |
|  | 11.2 Droughts |
|  | 11.3 Temperature Extremes |
|  | 11.4 Storms & Flooding |
|  | 11.5 Other Impacts |
| 1. **Unknown**   The threats classification scheme is intended to be comprehensive, but as there are often new and emerging threats, this option allows for these new threats to be recorded. | |
| 12.1 Other Threat | |
